# The Chemistry Behind ADCs

**DOI:** 10.3390/ph14050442

**Published:** 2021-05-07

**Authors:** Vesela Kostova, Patrice Désos, Jérôme-Benoît Starck, Andras Kotschy

**Affiliations:** 1 Drug Design Small Molecules Unit, Institut de Recherches Servier, 125 Chemin de Ronde, 78290 Croissy-sur-Seine, France; vesela.kostova@servier.com (V.K.); patrice.desos@servier.com (P.D.); jerome.starck@servier.com (J.-B.S.); 2 Servier Research Institute of Medicinal Chemistry, Zahony u 7, H-1031 Budapest, Hungary

**Keywords:** antibody-drug conjugates, linker, conjugation, attachment point

## Abstract

Combining the selective targeting of tumor cells through antigen-directed recognition and potent cell-killing by cytotoxic payloads, antibody-drug conjugates (ADCs) have emerged in recent years as an efficient therapeutic approach for the treatment of various cancers. Besides a number of approved drugs already on the market, there is a formidable follow-up of ADC candidates in clinical development. While selection of the appropriate antibody (A) and drug payload (D) is dictated by the pharmacology of the targeted disease, one has a broader choice of the conjugating linker (C). In the present paper, we review the chemistry of ADCs with a particular emphasis on the medicinal chemistry perspective, focusing on the chemical methods that enable the efficient assembly of the ADC from its three components and the controlled release of the drug payload.

## 1. Introduction

**Figure 1:**
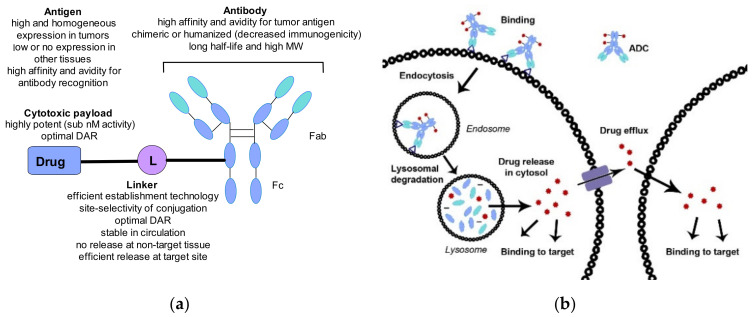
(**a**) The general structure of an ADC and the key considerations when combining the different components. (**b**) Schematic representation of the uptake of the ADC and the release of the payload inside a cancer cell.

Cancer is a leading cause of death worldwide and most of the existing therapies use small molecules (chemotherapy) to eradicate the cancer cells. Although they are widely applied, the use of most chemotherapies is limited by undesired side effects, mostly through action on cells beyond the tumor and its environment, resulting in systemic toxicity and a narrow therapeutic window. The discovery of the unique composition of cancer cell surfaces combined with the understanding of the strong and selective interaction between antibodies and cell-surface antigens opened the way to exploit antibodies as targeted delivery agents for chemotherapies, including highly toxic drugs [1,2,3]. The resulting molecular entities, also known as antibody–drug conjugates (ADC) consist of three main parts: the antibody responsible for the selective recognition of the cancer cell surface antigen capable of internalizing the ADC, the drug payload responsible for killing the cancer cell once released inside it, and the linker connecting the antibody and payload parts. Figure 1a shows the general structure and key features of an ADC, which will be reviewed in more detail later. Figure 1b depicts the key steps of an ADCs action. Recognition of the antigen by the ADC leads to a strong binding that initiates internalization through the endocytosis pathway [4]. Following lysosomal degradation, the payload is released in a biologically active form and exerts its effect, typically leading to the death of the cancer cell. The amount of the payload in the cytosol is determined by the number of surface antigens per cell, the number of drug payload molecules per ADC (aka drug-antibody ratio, DAR), and the time it takes for the return of the antigen on the cell surface [5]. The payload might escape the cancer cell either following its death and degradation or traversing its membrane from the cytosol. Consequences of this release can be beneficial (e.g., killing neighboring cancer cells, also known as the bystander effect), or detrimental leading to systemic toxicity. Therefore, the optimal drug payload properties for ADC use might differ from that of systemic use.

The simplified picture presented in Figure 1b might lead to the false understanding that taking an antibody and a toxic payload connected through some simple linker would give a drug. The facts that the first ADC (Mylotarg) was approved in 2000 (and following withdrawal in 2010 reapproved in 2017) and that the second ADC (Adcetris) received accelerated approval in 2011 and full approval in 2015 (Table 1) suggest otherwise. The third (Kadcyla) and fourth (Besponsa) ADCs were approved in 2013 and 2017, respectively. The slow start is proof of a long and complex learning phase, but the sustained efforts of the pharmaceutical industry seem to bear fruit. Since 2019, the number of approved ADCs more than doubled with five approvals in 2019–2020, signifying a better understanding of both the biology and chemistry of ADCs. Although in the present review we focus on conjugates bearing small molecules, it is important to note that conjugates with peptide toxins are also of therapeutic value exemplified by the approved drugs listed at the end of Table 1.

There are several changing parts in an ADC and apparently there is no generic formula to succeed. Thus, questions remain concerning: how to select the right antibody, where and how to attach the linker to the antibody, what kind of chemical linker to use to connect the antibody to the drug payload, how many drug molecules to attach per antibody, how to connect the linker and the drug payload, and what is the optimal drug payload like? Presuming that the biological background is robust, if we want to succeed in ADC development, we need to get the answer to all the above questions right, resulting in a unique combination of A-antibody, D-drug payload, and C-linker. In the following chapters, we review some of the key challenges and recent learnings focusing on the diverse aspects of ADC development where chemistry plays an important role.

## 2. ADC Payloads and Their Attachment to the Linker 

Payload is the key component of ADC to exert cytotoxic effects, which has to meet the following principal requirements: (1) highly cytotoxic, usually with the IC_50_ value at low nanomolar or picomolar level, (2) well defined target and action mechanism, (3) (potential) chemical attachment site. This third point is reviewed in this chapter, from a medicinal chemist point of view and highlights efforts of many teams to generate highly potent payloads with a suitable point of attachment for the linker, enabling bioconjugation with the mAb. Payloads are discussed by their mode of action divided into chemical families. For the sake of clarity, the manuscript uses a color coding: drug payloads are shown in blue, linkers in black, and the linkage site is highlighted by a yellow circle.

In this chapter we discuss only small molecule drug payloads, which usually exert their action through a well-defined biological mechanism. When reviewing the ADC literature, one has to acknowledge the significant achievements in using payloads beyond this chemical space, namely the attachment of radioactive nuclei through chelating groups (e.g., **12** incorporating a modified diethylenetriamin pentaacetic acid yttrium binder), and peptide toxins such as the 38 kDa Pseudomonas exotoxin fragment in **10**, or the Diphtheria toxin in **11** consisting of over 500 amino acid residues. Although not discussed in detail in this review, representative examples of such ADCs that reached later stage clinical development will also be summarized in Section 5.

### 2.1. Microtubule-Disrupting Drugs

#### 2.1.1. Auristatins

**Figure 2:**
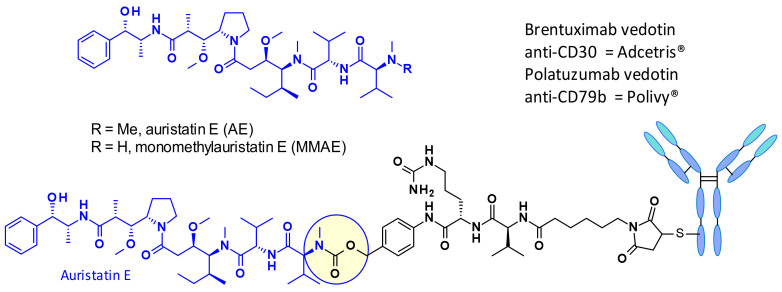
Structure of auristatine E (AE), monomethyl auristatin E (MMAE), and commercially approved auristatin-based ADCs: Target Antigen.

Auristatins are important payloads used in ADCs, and the most well-known family member, MMAE, is present in two marketed drugs, Adcetris© (**2**) and Polivy© (**5**, Figure 2). Currently, over 10 ADCs with auristatins such as MMAE or monomethyl auristatin F (MMAF) as payload are in clinical trials (see Section 5) [6].

**Figure 3:**
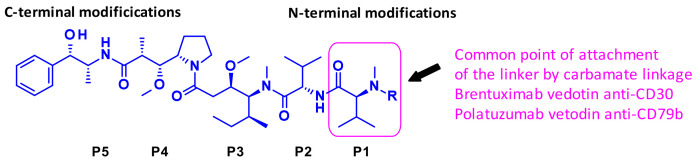
Auristatine terminal amino acid sites: N-terminus, P1 and C-terminus, P5.

**Figure 4:**
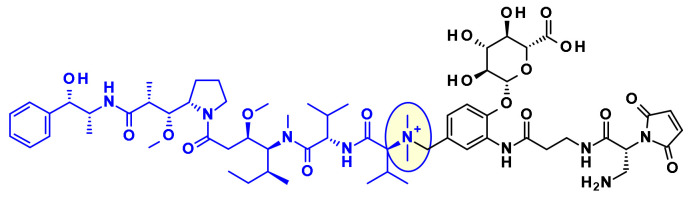
Seattle Genetics’s linker-payload combination including *N*-dimethyl auristatine and an ammonium linkage.

Figure 3 depicts auristatine and its frequently used linkage sites. The structure-activity relationship (SAR) of auristatins has been extensively studied focusing on the terminal subunits: P1 (N-terminus) and P5 (C-terminus) [7]. The most common approach is to introduce a carbamate functionality on P1 between the secondary nitrogen of auristatine and a cleavable ValCit Paba linker like in **2** and **5**. In 2015, researchers at Seattle Genetics expanded the scope of ADC payloads to include tertiary amines, and *N*-dimethyl auristatine in particular [8]. For the first time, the authors conjugated the drug to mAbs through an ammonium linkage (Figure 4). The resulting ADCs were stable under physiologic conditions, highly active in vitro and in vivo, and immunologically specific. These results expanded the repertoire of drugs that can be utilized within ADCs for targeted drug delivery to cancer cells.

**Figure 5:**
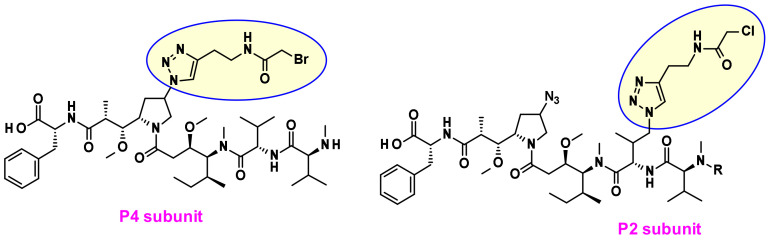
Novel drug linkers containing P2 or P4 linkage.

Recently Agensys modulated the central subunits P2-P3-P4 and generated novel hydrophilic derivatives with improved in vitro and in vivo potency when conjugated with protease-cleavable linkers [9] Introduction of azide groups into P2 and P4 subunits opened the way to serve as handles for linker attachment. Thus, novel drug–linker conjugates were prepared using click chemistry (Figure 5) and the payloads incorporating azide groups could serve as attachment sites for other non-cleavable or cleavable linkers.

**Figure 6:**
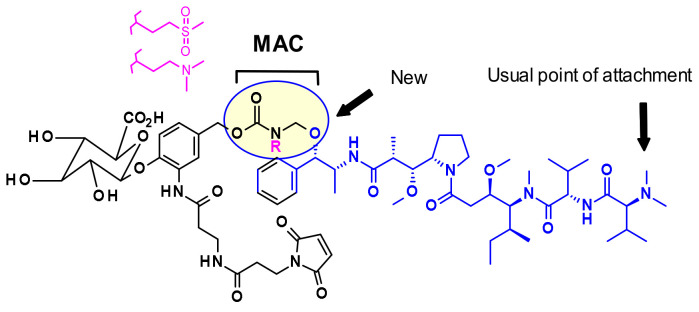
Methylene alkoxy carbamate (MAC) self-immolative unit for alcohol conjugation.

In general, in auristatins containing both amine and alcohol functions, the preferred point of attachment is the amine through a carbamate linkage. Seattle Genetics developed a new strategy for the bioconjugation of alcohol-containing payloads to antibodies that involves the methylene alkoxy carbamate (MAC, Figure 6) self-immolative unit [10]. To extend the MAC technology for ADCs, auristatin (AE) was incorporated through the norephedrine alcohol moiety. To stabilize the MAC linkage, both basic and electron-withdrawing groups are proximal to the aminal linkage. As a result, the conjugates were stable under physiological conditions, demonstrated high potency, and were immunologically specific both in vitro and in vivo.

**Figure 7:**
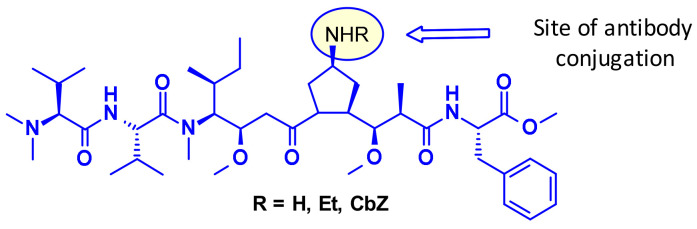
Structure of azastatins.

Recently, researchers at Uppsala University introduced azastatins (Figure 7), as a new class of potent auristatin derivatives encompassing a central amine side chain for antibody conjugation. Their results establish these auristatine derivatives as a novel class of cytotoxic payloads suitable for ADC development [11].

#### 2.1.2. Maytansinoid Derivatives (DM2, DM4)

**Figure 8:**
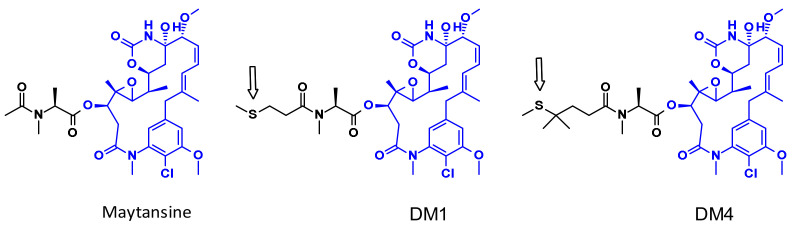
The structure of maytansine and its conjugable derivatives DM1 and DM4.

Maytansine is a very potent inhibitor of microtubule assembly inducing mitotic arrest in intoxicated cells. This structure is difficult to conjugate because it doesn’t have reactive functional groups. To overcome this problem, a series of very potent derivatives containing an SMe group (as prodrugs of SH liberated through a reduction process by glutathione following cellular uptake) were created. First examples of this class are DM1 and DM4 (Figure 8) bearing a methylthiopropanoyl group instead of the native *N*-acetyl group [12].

**Figure 9:**
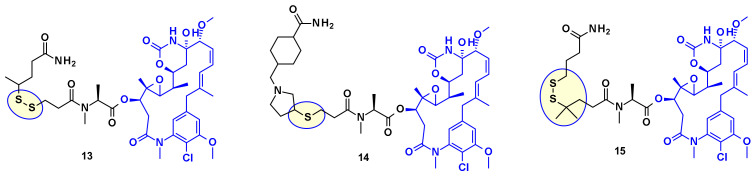
Chemical linkage of DM1 and DM4 through a disulfide or sulfide bond.

From the payloads DM1 and DM4 several ADC precursors have been synthesized by employing a disulfide linkage (Figure 9) with either a labile disulfide bound as in compound **13**, a hindered disulfide bound as in compound **15**, or a stable thioether bound as in compound **14**. The stabilized disulfide linker showed a good stability in blood circulation while maintaining an efficient cleavage inside the cell.

**Figure 10:**
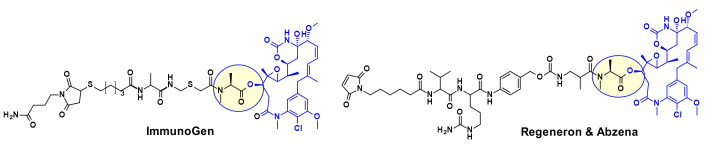
Maytansine-derived drug-linker precursors.

Several maytansine-based ADCs were prepared using the same secondary hydroxyl group as attachment point and carrying in most cases a linker for transglutaminase bioconjugation. For example, a Daratumumab (CD38 mAb approved by the FDA for the treatment of multiple myeloma) based ADC was shown to deliver DM4 specifically to CD38 overexpressing cancer cells [13]. ImmunoGen prepared recently a new type of ADC that contains a sulfur-bearing maytansinoid (Figure 10) attached to an antibody via a highly stable tripeptide linker [14]. The attachment point is the same hydroxyl group as above. The authors showed that increasing the number of methylene units in the linker increased bystander killing activity and improved efficacy in vivo in mice compared to previously described maytansinoid conjugates. In a similar approach, keeping the core macrocycle unchanged, researchers at Regeneron and Abzena investigated the effect of substitution at the *N*-methyl alanine nitrogen also varying the length of the side chain on the macrocycle, as well as the linker attachment through a primary versus secondary amine [15]. The best results were found when the precursor shown in Figure 10 was bioconjugated with EGFR.

#### 2.1.3. Tubulysins

**Figure 11:**
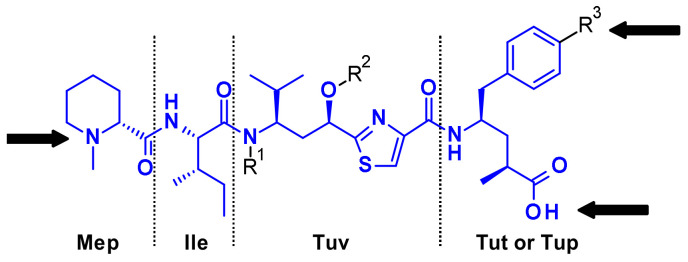
Structure of Tubulysins and principal points of attachment.

Tubulysins are potent inhibitors of microtubule polymerization, causing rapid disintegration of the cytoskeleton of dividing cells, and leading to apoptosis [16]. They are a family of naturally occurring tetrapeptides, containing *D*-*N*-methyl-pipecolic acid (Mep), *L*-isoleucine (Ile), tubuvaline (Tup) and either tubutyrosine (Tut, R^3^ = OH) or tubuphenylalanine (Tup, R^3^ = H) (Figure 11).

**Figure 12:**
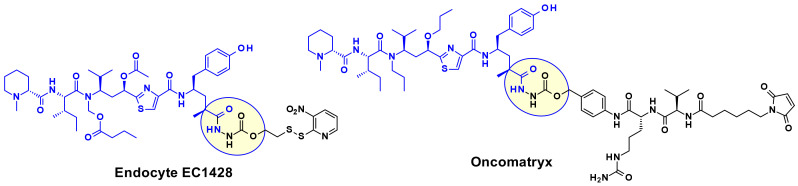
Examples of Tut attached Tubulysin payloads.

A wide range of attachment points have been developed for the utilization of tubulysins as ADC payloads [17]. An obvious point of attachment in such a structure is the carboxylic acid of the Tut or Tup building block. This approach is exemplified by Endocyte’s tubulysin disulfide linker -payload EC1428 (Figure 12)**,** where the carboxylic acid was connected to the linker through a hydrazide moiety [18]. The same approach has been used by Oncomatryx where a cleavable PABAValCit maleimide linker was installed the same way [19].

Another approach used by AstraZeneca [20], Bristol-Myers Squibb [21], and Pfizer [22] relies on the derivatization of the phenyl ring in Tup or Tut to produce an attachment handle through the introduction of amino function (Table 2). 

**Figure 13:**
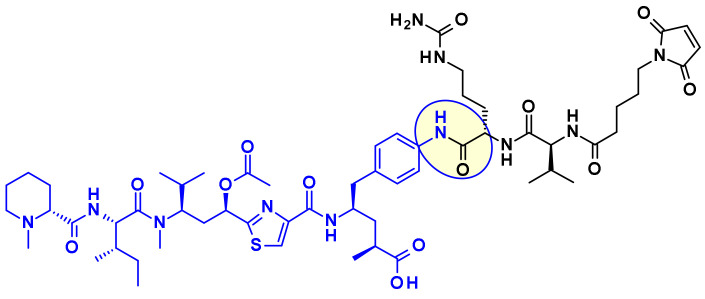
Examples of phenyl attached tubulysin linkers payloads.

Very recently Bristol Myers Squib researchers used this same amino handle to introduce a Cit-Val dipeptide linker via an amide bond (Figure 13) [23].

**Figure 14:**
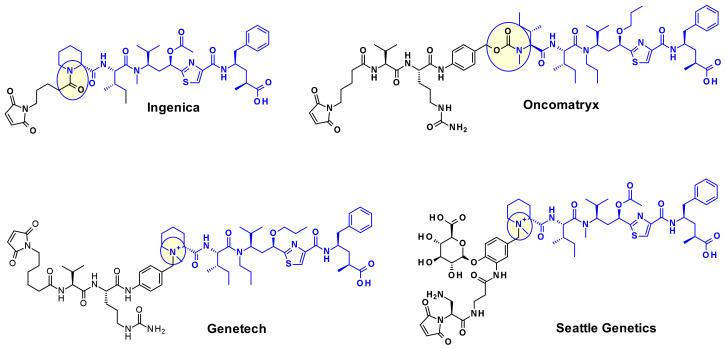
Examples of Tubulysin payloads attached to linkers through the Mep group.

The attachment of linkers to the Mep group has also been studied extensively. Ingenica researchers reported that the des-methyl Mep analog retains potent cytotoxic activity, and could be considered as valuable payload, allowing the introduction of a non-cleavable maleimide caproyl linker onto the secondary amine (Figure 14) [24,25,26]. Oncomatryx showed that the introduction of a cleavable linker via a carbamate linkage is an efficient approach to produce ADCs when the Mep is replaced by another motif conserving a secondary amine. Particularly interesting is the report by Genentech about attaching traceless linkers to tertiary amine containing payloads via quaternary ammonium groups [27]. The introduction of mc-Val-Cit-PABA linker on the payload led to more hydrophilic conjugates and improved stability in the blood. The same approach was used by Seattle Genetics with a glucuronide linker that also provides improved hydrophilic properties and selective intracellular cleavage by over expressed β-glucuronidase in cancer cells [8].

#### 2.1.4. Cryptomycins

**Figure 15:**
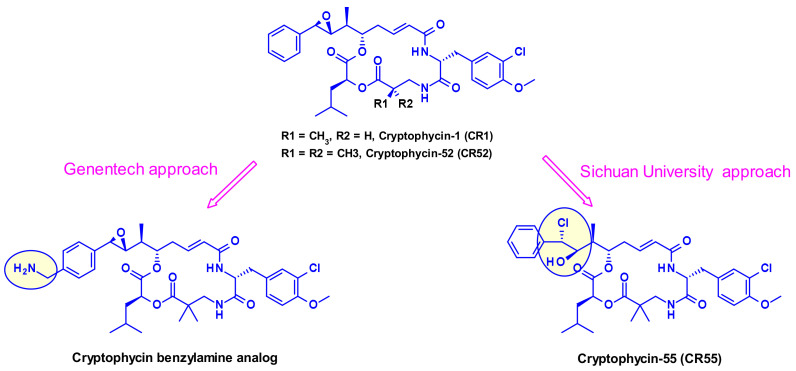
Two approaches for introducing handle in Cryptomycin.

**Figure 16:**
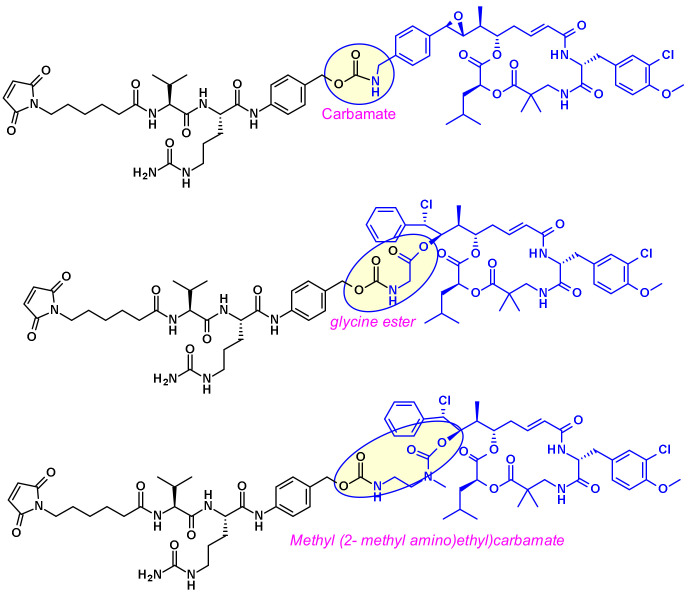
Examples of cryptophycin-derived linker-payload precursors.

Cryptomycins (CR) are a family of 6-membered macrocyclic depsipeptides displaying potent activity against cancer cell lines, which bind to microtubules at the vinca binding site. Significant efforts were put forth to advance this class of compounds clinically but results from clinical trials indicated unacceptable levels of toxicity at doses required for a therapeutic effect. Several groups worked on their vectorization using the ADC format. Due to the lack of a coupling site in CR two distinct approaches were utilized, first by Genentech [28] and recently by the Shinchua University [29] both aiming at the introduction of a biocompatible functionality enabling the attachment of the linker for ADC construction. Genentech researchers converted benzene to a benzylamine to produce a potent payload, which is suitable for vectorization via a carbamate linkage (Figure 15). In the second approach, the authors took advantage of the prodrug form of cryptophycin-52 (CR55, Figure 15) that can re-cyclize to CR52 under physiological conditions. Selected examples of cryptophycin-derived linker-payloads are shown in Figure 16.

#### 2.1.5. Antimitotic EG5 Inhibitors

**Figure 17:**
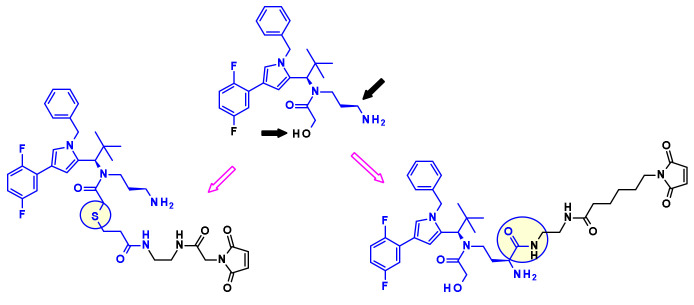
Identification of linker attachment points of the pyrrole based KSP inhibitor and examples of linker-payload conjugates prepared by Bayer.

Kinesin spindle protein (KSP, also known as Eg5 or KIF11) is an ATP-dependent motor protein involved in the separation of centrosomes of the cell cycle [30]. Thus, blockade of this essential event in mitosis with KSP inhibitors (KSPis) results in high antitumor potency. Bayer discovered a new pyrrole subclass of KSPis represented by the structure shown on Figure 17 [31]. They investigated different positions of the molecule that are compatible with the attachment of linker retaining the strong affinity to KSP. The established SAR revealed two appropriate sites, marked by black arrows in Figure 17.

**Figure 18:**
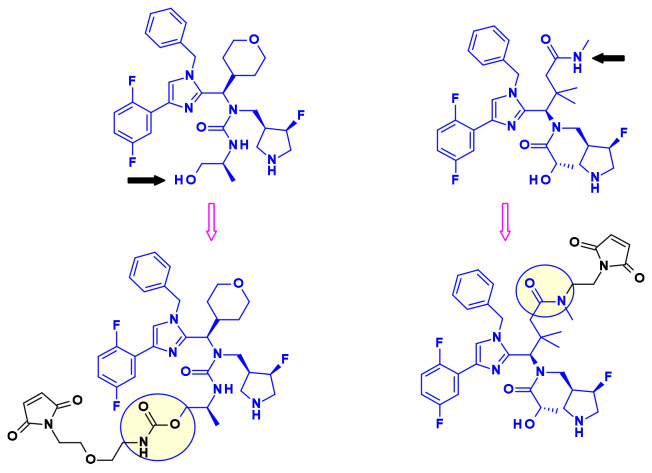
Vectorization of imidazole-based KSP inhibitors.

In a similar approach, researchers at Novartis started from imidazole containing KSP inhibitors (Figure 18) as starting points in their Eg5 ADC effort. Using the primary alcohol or the secondary amide moieties they installed non cleavable linkers with a maleimide end group [32]. When coupled with antibodies targeting HER2 and c-KIT the resulting ADCs demonstrated superior in vivo efficacy compared to ado-trastuzumab emtansine (Kadcyla, **3**).

### 2.2. DNA Damaging Drugs

#### 2.2.1. Pyrrolobenzodiazepines and Indolinobenzodiazepine

**Figure 19:**
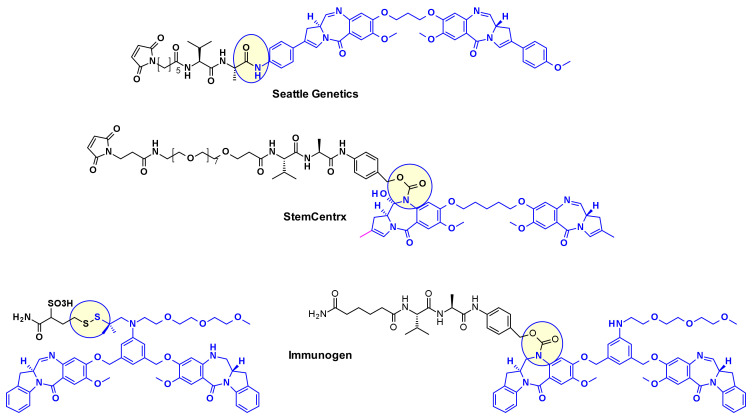
Attachment of linkers to PBD and IBD payloads.

Pyrrolo[2,1-*c*][1,4]benzodiazepines (PBD) fall within the class of natural products with antitumor activity. Their mode of action involves selective alkylation in the minor groove of DNA, where N2 of guanine forms a covalent bond with the electrophilic N10/C11 imine on the PBD. A comprehensive review of the development of PBD containing ADCs has recently been published by researchers at Femtogenix [33]. It describes the different strategies for linking PBD dimer payloads [34]: Seattle Genetics used the aniline of SGD1882 as attachment point mimicking the PAB unit commonly used in cleavable linkers and releasing the free PBD payload (Figure 19). StemCentrx in collaboration with Spirogen used the N-10 position of PBD to connect the linker thought a carbamate. The same carbamate linkage was also used in the structurally similar indolinobenzodiazepine dimer (IBD) payload by Immunogen [35]. They also reported a different approach for the same class of IBD where a substituted benzene ring was used as linker between the C8/C8’ positions of the two IBD monomers.

**Figure 20:**
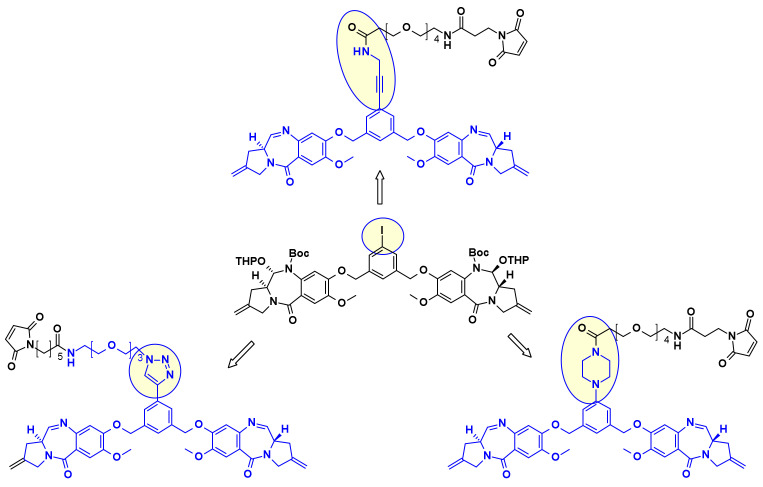
PBD dimer/linkers attachment using a common iodobenzene intermediate.

In a similar approach, Spirogen and Genentech designed an iodobenzene linked PBD, which allowed for the introduction of different linkers in transition metal catalyzed reactions [36]. By using Sonogashira-coupling, Buchwald–Hartwig coupling, or azide-alkyne click reaction alkyne, piperazine, or triazole linked linker-payload conjugates were respectively obtained (Figure 20).

#### 2.2.2. Duocarmycins

**Figure 21:**
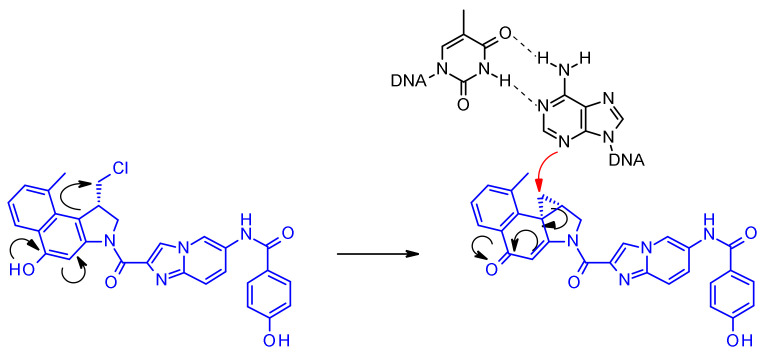
Activation of duocamycin derivatives and mechanism of action of alkylation of N3 adenine in the minor groove of DNA.

Duocarmycins are powerful cytotoxic substances and bind to the minor groove of DNA and alkylate the adenine at the N3 position through their highly reactive cyclopropane ring [37]. In the non-cyclized, halomethyl form duocarmycins present significantly reduced cytotoxic activity. Since the phenol function within the alkylating portion of the molecule can acts as a mesomere activator enabling the formation of the electrophilic cyclopropane (Figure 21), linking strategies in the development of duocarmycin ADCs focused on linker attachment of the phenolic functional group, whose liberation led to the generation of the active form.

**Figure 22:**
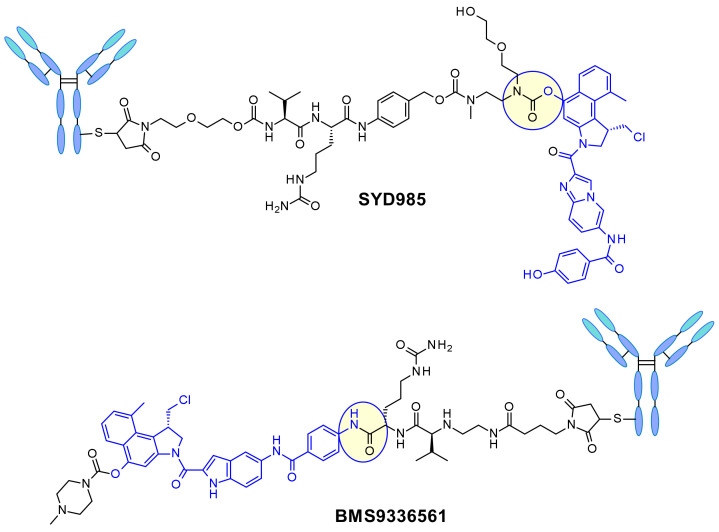
Structure of the ADCs developed by Synthon (SYD985) and Medarex (BMS9336561).

In SYD985, developed at Synthon, the phenol group is the site for connecting the linker through a double carbamate to the Mc-val-cit-PABC unit (Figure 22) [38]. After cathepsin B cleavage, the free phenol promotes intramolecular rearrangement to the electrophilic cyclopropyl form. Medarex employed a different approach attaching the linker via an aromatic amine in the non-alkylating part of the molecule and masking the phenol prodrug with an *N*-methyl piperazine carbamate moiety [39]. In this case, the phenol will be liberated and subsequently the active cyclopropane will be formed under the action of a carboxylesterase.

#### 2.2.3. Camptothecin

**Figure 23:**
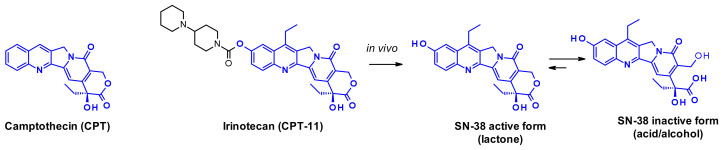
Structure of camptothecin, irinotecan and its metabolite SN-38.

Camptothecin (CPT) and its derivatives are classical examples of topoisomerase I inhibitors [40]. They stabilize the transient single-strand DNA break due to topoisomerase-induced cleavage, and when the ternary DNA-TOP1-inhibitor complexes encounter the replication fork, double-strand cleavage of DNA occurs. The natural alkaloid camptothecin is a pentacyclic structure whose extreme insolubility prevents widespread use as a cancer therapeutic. Its water-soluble prodrug, irinotecan gained marketing approval for metastatic colorectal cancer. SN-38 is the active metabolite of Irinocetan, generated in vivo by the action of human liver carboxylesterase (Figure 23) that can be inactivated by the opening of the lactone ring.

**Figure 24:**
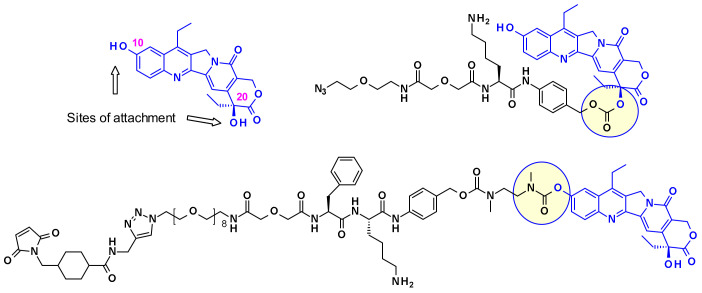
The vectorization points of SN38 and representative linker-payload conjugates.

Immunomedics established two different strategies to conjugate SN-38 through one of its hydroxyl moieties (Figure 24). In one example, the linker is attached through the more reactive C-10 phenol group giving a stable carbamate bond, while in the other example through the C-20 hydroxyl group while stabilizing the lactone form [41], which is crucial for in vivo potency.

**Figure 25:**
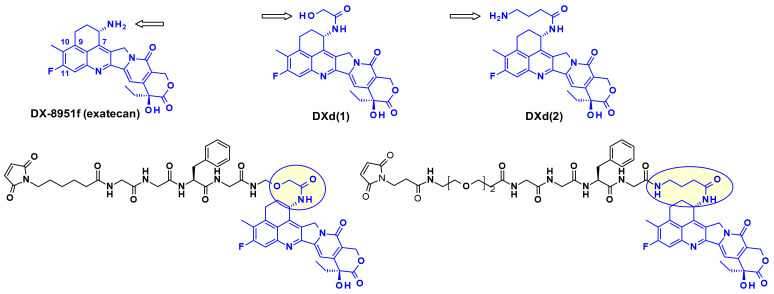
Structures of exatecan and its derivatives, DXd(1) and DXd(2), as well as the derived conjugates.

Another very potent drug, suitable for ADC construction, is exathecan (DDX-8951f) (Figure 25) [42]. This camptothecin analog possesses an amine substituent on its cyclohexane ring, bridging the 7 and 9 positions [43]. The amino group of exatecan contributes to its water solubility, while the rigidity conferred by the cyclohexane ring is thought to favor the equilibrium towards the active lactone form versus the inactive hydrolyzed hydroxy acid. Building from the amino group hydroxy acetylation led to DXd(1) while 4-aminobutyroylation gave DXd(2), both compounds retaining the biological activity of Exatecan. 

The pendant hydroxyl and amino groups were obvious attachment points to vectorize the payloads using enzyme-cleavable Gly-Gly-Phe-Gly tetrapeptide linkers (Figure 25). The ADCs resulting from bioconjugation to an anti-HER2 antibody showed great potential against HER2-expressing cancers in the clinical setting [44].

**Figure 26:**
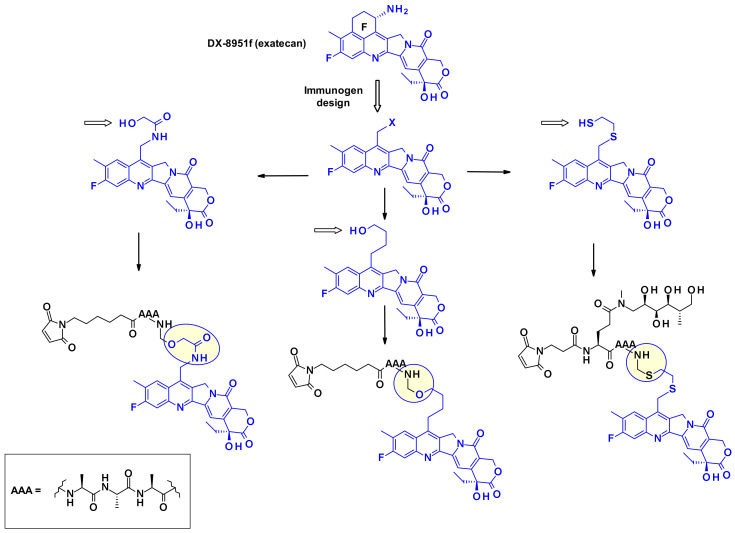
Elimination of the ring F by Immunogen and design of new related camptothecin L/P.

The cyclohexylamine ring of DXd, although supposedly stabilizes the bioactive lactone form, carries a chiral center that complicated the synthetic efforts and SAR studies (Figure 26). To overcome this difficulty researchers at Immunogen investigated a new set of camptothecin analogs capable of being conjugated to a mAb, where this ring was opened-up and the additional chiral center was eliminated [45]. Starting from a common intermediate, the authors prepared three types of handles (Figure 26) and subsequently vectorized the payloads using various polyalanine linkers. When bioconjugated to anti-HuEGFR, the resulting ADCs were efficacious on EGFR-positive HSC-2 carcinoma xenografts.

#### 2.2.4. Calicheamicin

**Figure 27:**
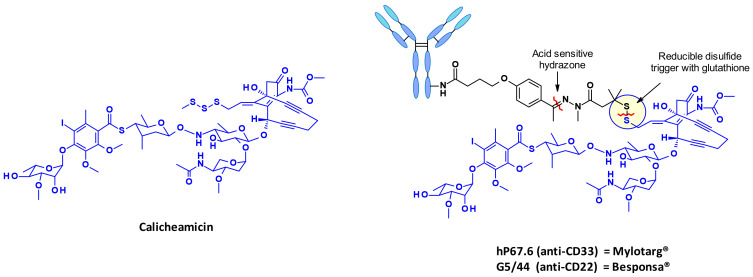
Structure of calicheamicins and the design of the two marketed ADCs Mylotarg^®^ and Besponsa^®^.

Calicheamicins constitute a very well-studied class of enediyne antibiotics with a particularly intriguing and complex structure and mechanism of action which make them a class of their own in the field of ADC payloads. Strategies for linking calicheamicin in ADCs are well exemplified by the marketed ADCs Mylotarg^®^ and Besponsa^®^ (Figure 27). The payload release proceeds in two steps: a sensitive cleavage of the hydrazone in the acidic intracellular environment, followed by the reduction of the disulfide bond by intracellular glutathione.

**Figure 28:**
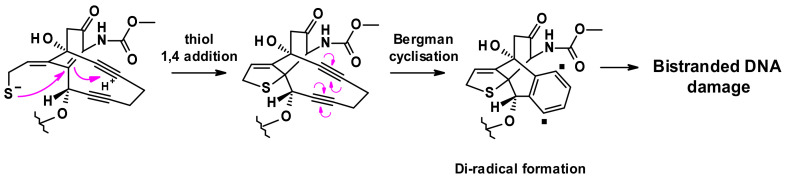
Mechanism of di radical formation from activated calicheamicin.

The released thiolate undergoes intramolecular 1,4-addition to the enone triggering a Bergman cyclisation that produces a di-radical. This reactive intermediate is capable of abstracting hydrogen atoms from the deoxyribose backbone to produce double strand DNA breaks, which in turn result in cell death (Figure 28) [46].

**Figure 29:**
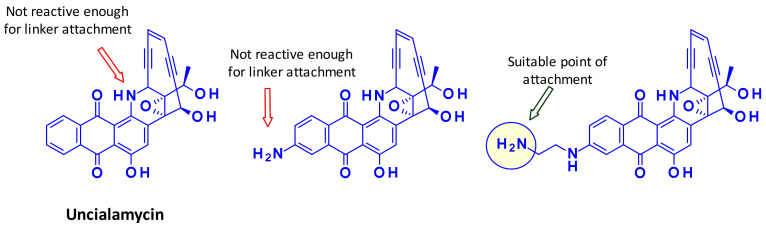
Research of a suitable handle on Uncialamycin.

Recently, a new enediyne natural product called Uncialamycin was isolated from Streptomyces found in lichen of British Columbia. The structure was confirmed by total synthesis and since then several highly potent synthetic analogues were prepared as potential payloads for ADCs [47,48]. Researchers at BMS showed that the secondary amine of Uncialamycin is not a suitable linker attachment point due to its low reactivity under various peptide coupling conditions. The authors synthesized an analog where an amino group is introduced directly onto the aromatic ring, but the reactivity of this aniline was also too weak to serve as handle for linker introduction. Installing an aliphatic amine on the other hand, using an aminoethyl extension, provided a suitable attachment point for linkers (Figure 29).

**Figure 30:**
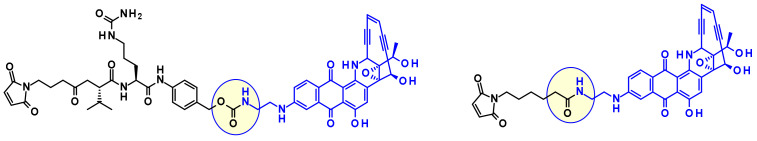
Cleavable and non-cleavable linker-payload conjugates.

From the latter payload they prepared precursors using both protease-cleavable dipeptides and non-cleavable linkers (Figure 30). Whereas the CD70 ADC having a cleavable linker showed highly specific cytotoxic activity on renal cell carcinoma cell lines, the corresponding non-cleavable ADC was inactive on the same cell lines. 

**Figure 31:**
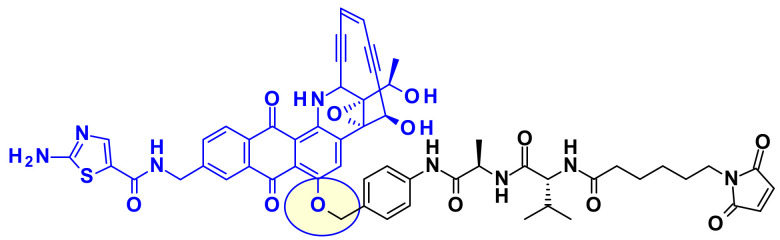
Linking of an uncialamycin analog through its phenol group.

Recently, in the continuation of this work, researchers at Bristol-Myers Squibb used the phenol group as point of attachment in designed, highly potent, and chemically stable Uncialamycin analogues (Figure 31) [49]. The authors used newly developed phenol alkylation chemistry to append a classical cleavable linker to the phenol handle of the payloads. Conjugation of the resulting payload with appropriate antibodies provided ADCs, which showed antigen-specific antitumor activity both in vitro and in vivo.

### 2.3. Innovative Drugs

#### 2.3.1. Apoptosis Inducers (Bcl-x_L_ Inhibitors)

**Figure 32:**
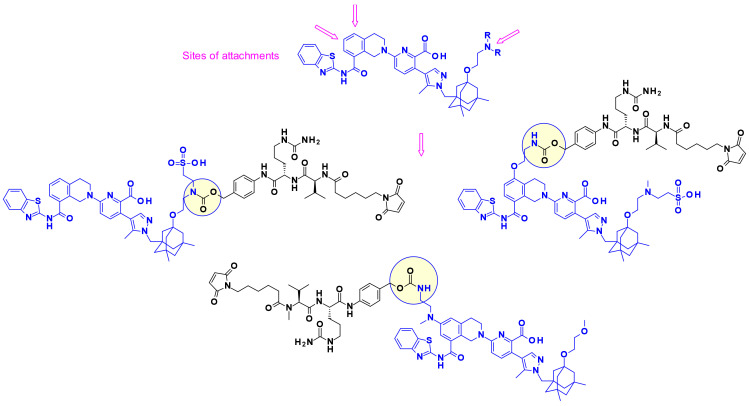
Attachment sites on a typical Bcl-xL inhibitor and some reported payload-linker conjugates.

One of the mechanisms by which cancer cells acquire resistance to apoptosis is the over expression of antiapoptotic Bcl-2 family members, including Bcl-x_L_. Agents capable of blocking the BH3-binding domain present on Bcl-x_L_ were shown to trigger apoptosis in cancer cells. In 2017, AbbVie disclosed the first vectorization of BcL-x_L_ inhibitors in the form of an ADC, which targeted specific cells or tissues that express EGFR [50,51]. Interestingly, the authors used three different points of attachment on the payloads to append cleavable linkers (Figure 32). Core modifications by aminoalkyl extensions were used to establish suitable linking sites where needed.

#### 2.3.2. Thailanstatin and Analogues

**Figure 33:**
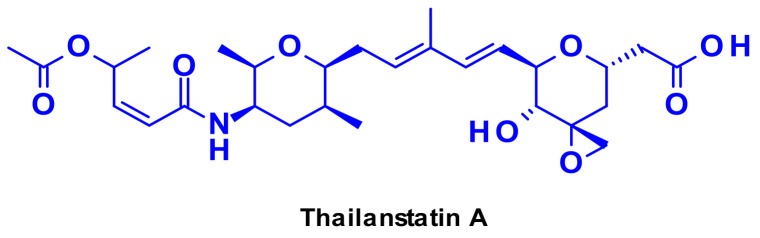
Structure of thailanstatin A.

Targeting the spliceosome, a large ribonucleoprotein complex involved in mRNA processing, offers a promising therapeutic option for targeted cancer therapy [52]. There are several natural products capable of inhibiting RNA splicing through binding to different spliceosome subunits [53]. The most representative is thailanstatin A (Figure 33), which can bind to the SF3b subunit of the spliceosome, thus preventing RNA splicing.

**Figure 34:**
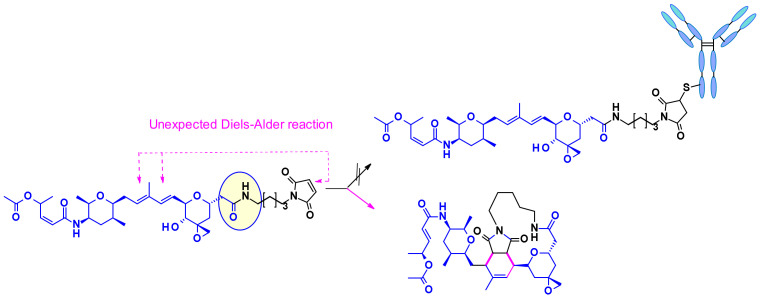
Unexpected Diels Alder reaction between the diene present in the thailanstatine structure and bioconjugation motif, maleimide.

**Figure 35:**
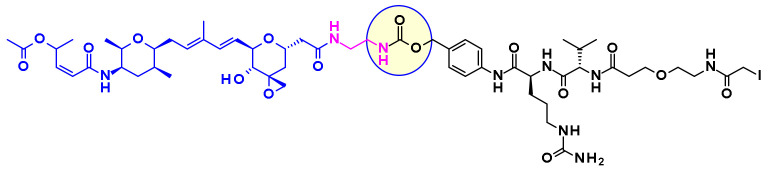
Thailanstatine-based payload-linker conjugate bearing amine spacer on the carboxylic acid and an iodo acetamide end group to avoid intramolecular cyclisation.

Thailanstatin A lacks a suitable handle for linker attachment. To address this issue the carboxylic acid was coupled with an ethylene diamine to introduce an amine-containing spacer, which is frequently used for the installation of linkers (Figure 34) [54]. Another difficulty to vectorize this natural product is the presence of multiple reactive functionalities. For example, the diene in the central core can react with the maleimide moiety used for bioconjugation, through a Diels–Alder reaction (Figure 34). This problem was addressed by using an alternative conjugating moiety, a halo-acetamide. The ADCs, combining those two modifications and containing a cleavable linker were first reported in the patent literature (Figure 35), and they are claimed to have modest activity in several HER2 expressing cell lines [55].

**Figure 36:**
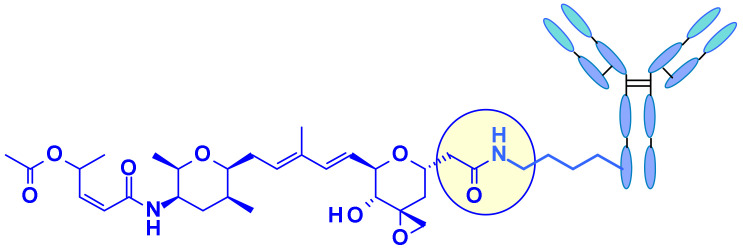
Pfizer’s “linker less” thailanstatin ADC.

Recently, Pfizer collaborators reported that the direct conjugation of the carboxylic acid to available surface lysines of the antibody (“linker less” conjugate) resulted in the most potent thailanstatin ADCs to date (Figure 36) [54]. Activity of these lysine conjugates was correlated with drug-loading, a feature not typically observed for other payload classes. The ADCs displayed excellent potency in a gastric cancer xenograft model.

#### 2.3.3. Amatoxins

**Figure 37:**
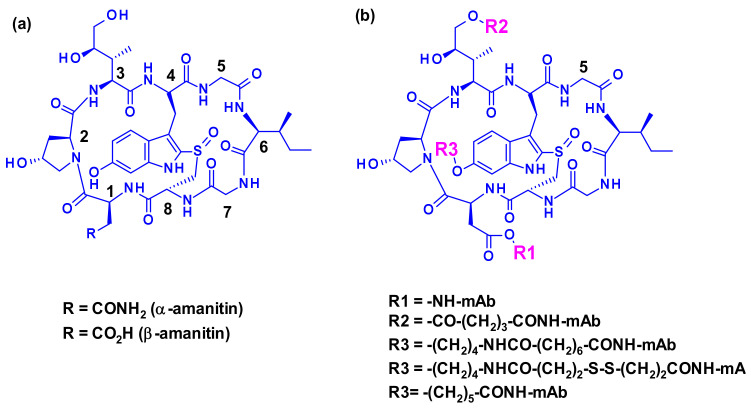
(**a**) Structures of the bicyclic octapeptide toxins α-amanitin and β-amanitin and their amino acid constituent numbering (**b**) Conjugation sites available in amatoxin for coupling to antibodies through linkers

The use of transcription inhibitors like amatoxins is a relatively new approach in the field of ADC technology. The nine naturally occurring amatoxin derivatives share the same skeletal structure, a macrocycle of eight *L*-configured amino acids, bridged between a tryptophan and a cysteine residue by a sulfoxide moiety (Figure 37). Three of the side chains in amatoxins are hydroxylated, the OH groups being responsible for good water solubility and binding to the target molecule. Two of the peptides, α-amanitin and β-amanitin, account for the 90% of all amatoxins.

Three attachment points were used on amatoxins to produce ADCs (Figure 37). The first attempts involved linker less conjugation of the β-amanitin carboxyl group to the amino group of IgG lysine. This led to conjugates with good plasma stability and high cytotoxicity on target-positive cancer cells but the yield of this bioconjugation was very low [56]. However, the introduction of a linker attached to this carboxyl group improved bioconjugation while maintaining stability and toxicity. The hydroxyl group of dihydro-isoleucine was also considered as a point of attachment. Introduction of a glutaric acid as a linker, followed by lysine conjugation led to an ADC with excellent in vitro cytotoxicity and in vivo antitumor activity, but unfortunately with poor stability in circulation due to the cleavage of the linker by serum carboxylesterase [57]. The third approach, attachment to the 6-hydroxyl group of the tryptophan represents the standard procedure for vectorization. Thus, etherification of the phenol with various linkers led to highly stable and potent ADCs. The others amino acids of amanitin (i.e., hydroxyproline, glycine, isoleucine, and cysteine) cannot be used for conjugation or linker attachment since they are either chemically unreactive and/or crucial for the binding of amanitin to RNA polymerase II.

**Figure 38:**
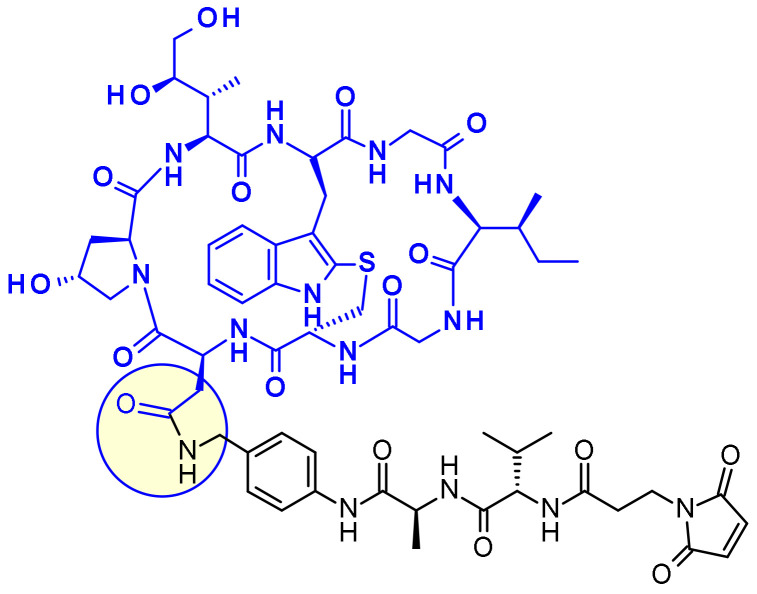
Structure of Heidelberg Pharma’s ADC precursor.

An advanced amanitin-based ADC is HDP-101 [58] (Figure 38). The payload itself is a synthetic amanitin derivative optimized for stability. The two differences compared to natural amanitin are the absence of the 6′-OH from tryptophan and the replacement of the sulfoxide by a thioether link. The cathepsin B-cleavable linker was introduced through amide formation on the aspartic acid side chain. 

**Figure 39:**
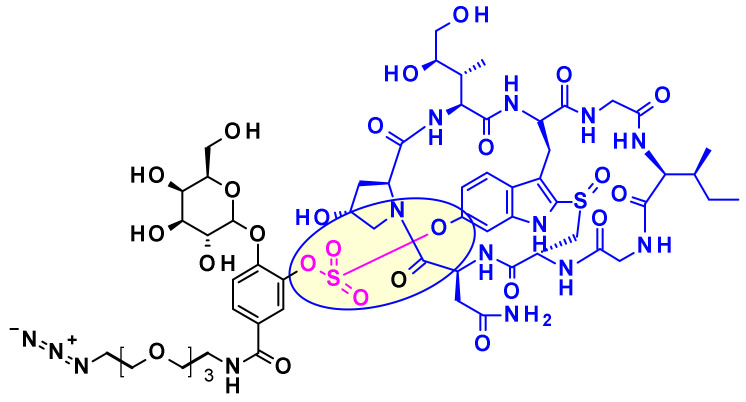
Structure of an OHPAS-linked α-Amanitin payload.

Recently Park and collaborators devised a new self-immolative linker motif (OHPAS) for phenol containing payloads (Figure 39) [59]. It is a diaryl sulfate, with one aryl moiety coming from the payload and the other from a latent phenol function at the ortho position of a self-immolative unit. This technology was applied to the vectorization of α-amanitin in trastuzumab ADCs, which exhibited potent in vitro and in vivo cytotoxicity.

#### 2.3.4. Inhibition of the Nicotinamide Phosphoribosyltransferase (NAMPT)

**Figure 40:**
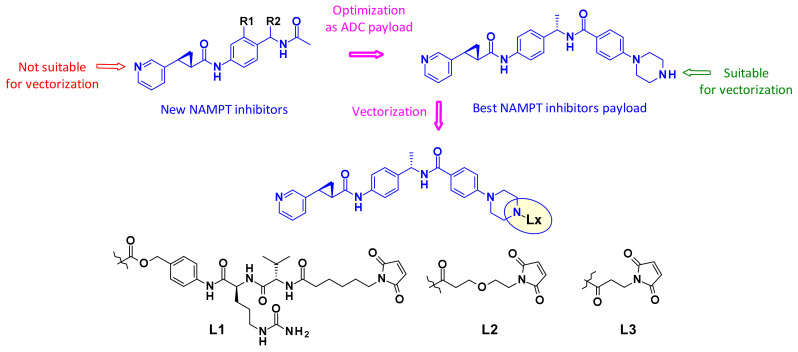
NAMPT inhibitors as payloads for ADCs.

Inhibitors of the nicotinamide phosphoribosyltransferase (NAMPT), an enzyme responsible for the conversion of nicotinamide to nicotinamide mononucleotide, showed efficacy in various preclinical and clinical studies, but their clinical utility has been limited by on-target and dose limiting toxicities such as thrombocytopenia and adverse GI effects. Therefore, researchers at Novartis looked for new NAMPT inhibitors that were suitable for vectorization as ADC payloads (Figure 40) [60]. Using NAMPT-inhibitor X-ray cocrystal structures they determined the suitable exit vectors for linking. The pyridine nitrogen was not considered as attachment point since it is known to undergo ribophosphorylation by the NAMPT enzyme and is thus an essential part of the pharmacophore. Optimization led to the introduction of a piperazine moiety to the payload at the para-position of the phenyl ring. This molecule displayed single digit nanomolar potency on c-Kit and HER2 expressing cell lines and was, therefore, selected for vectorization with a focused set of cleavable and non-cleavable linkers. The two non-cleavable linkers (L2 and L3) afforded ADCs that were generally low aggregating with very promising in vitro profiles. Furthermore, the conjugates were well tolerated and demonstrated target-dependent efficacy in vivo.

#### 2.3.5. Carmaphycins

**Figure 41:**
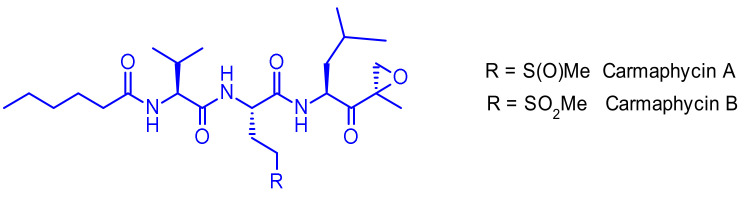
Structure of carmaphycin A and B.

Carmaphycin A and carmaphycin B, two new peptidic proteasome inhibitors were isolated as trace components from a Curaçao collection of the marine cyanobacterium *Symploca* sp. [61]. Both feature a leucine-derived α,β-epoxyketone warhead directly connected to either methionine sulfoxide or methionine sulfone (Figure 41). They were found to inhibit the β5 subunit (chymotrypsin-like activity) of the *S. cerevisiae* 20S proteasome in the low nanomolar range. Additionally, they exhibited strong cytotoxicity to lung and colon cancer cell lines.

**Figure 42:**
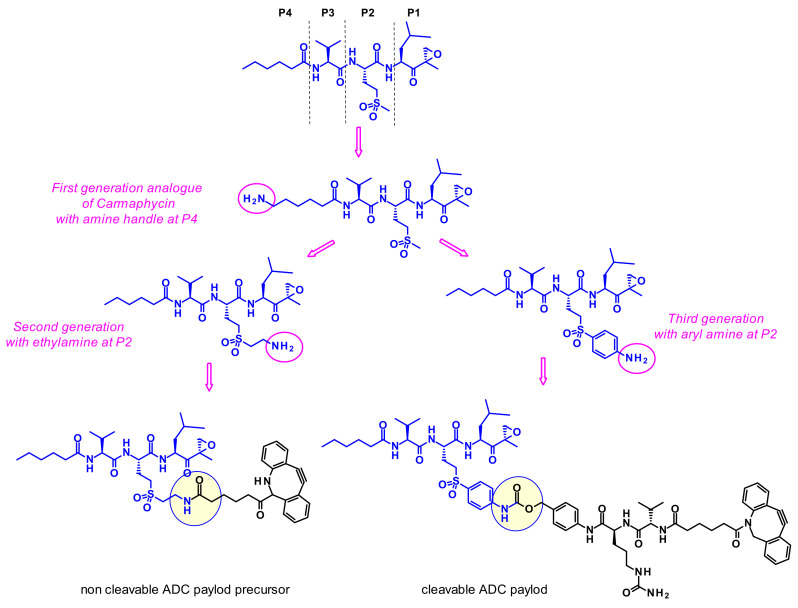
Evolution of carmaphycin B in order to install a suitable amine handle and examples of non-cleavable and cleavable ADCs precursors.

However, because of their high potency, their selectivity is suboptimal, and they frequently show toxic side effects. Gerwick and coworkers therefore hypothesized that utilizing a highly toxic carmaphycin derivative as the warhead of an ADC might maintain the required potency and achieve a better tolerance [62]. The designed analogues all incorporated sulfone methionine derivatives at the P2 position (like carmaphycin B) rather than the sulfoxide methionine as in carmaphycin A, as this eliminated structural complexity arising from the mixture of stereoisomers at the sulfoxide group (Figure 42). First generation analogues harbored an amine group at the distal P4 terminus. Unfortunately, this attachment point was not appropriate since the payloads displayed reduced cytotoxic activity. Second generation carmaphycin analogues contained an amine handle on the P2 side chain. The best results were obtained when the short ethyl amino chain extends the sulfonyl group. In third generation analogues an aryl group links the sulfone and the amine, therefore reducing its basicity. Both second and third generation payloads showed potent in vitro activities and were vectorized with cleavable or non-cleavable linkers. Unfortunately, none of the resulting ADCs exhibited such cell killing against the tested cancer cell lines that was superior to the free antibody trastuzumab.

## 3. Linker Types 

Linker is not only the molecular moiety making the covalent connection between the antibody and the small molecule payload, but also a key element in targeted drug therapy having designed properties. Its incorporation should not induce aggregations, ensure acceptable PK properties of the construct while limiting premature release of the payload in plasma (stability) and enabling efficient release of the active molecule at the targeted site of action. Schematically, linkers are divided into two categories: non-cleavable and cleavable.

### 3.1. Non-Cleavable Linkers

**Figure 43:**
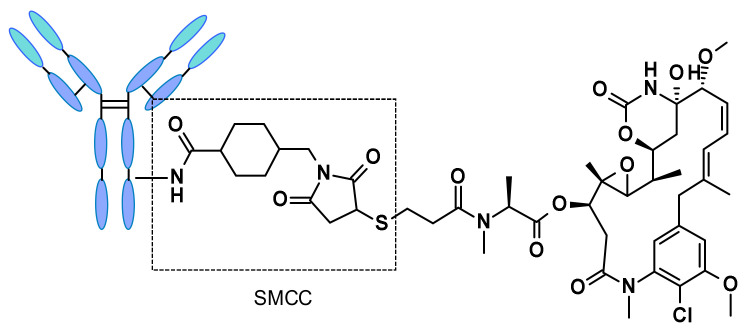
Structure of trastuzumab emtansine (Kadcyla^®^).

Non-cleavable linker-based ADCs must be internalized, and the antibody part needs to be degraded by lysosomal proteases to release the active molecule. Many non-cleavable linkers have been explored in ADC development, the most representative being *N*-succinimidyl-4-(*N*-maleimidomethyl)cyclohexane-1-carboxylate (SMCC), present in trastuzumab emtansine (Figure 43). Catabolism of such constructs led to Lys-SMC-DM1 as the major tumor metabolite [63]. Furthermore, drugs linked to such linkers usually cannot exert by-stander effect [64], since the catabolites released have poor permeability [65]. The present research focuses mainly on cleavable linkers, and considerable effort is invested in the design of molecular entities capable of triggering the efficient release of well-defined and characterized payloads.

### 3.2. Cleavable Linkers

The use of cleavable linkers is equally viable for the design of internalizing and not internalizing ADCs, because the release is triggered by the nature of the cleavage site (lysosome and/or tumor environment). Linkers can be separated into two major categories: enzymatically and chemically (i.e., non-enzymatically)-labile.

#### 3.2.1. Non-Enzymatic Linkers

**Figure 44:**
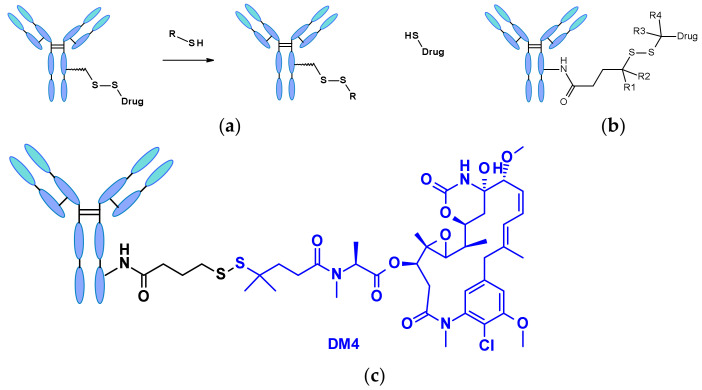
(**a**) Schematic structure of disulfide-containing linker and its reaction with thiols, like GSH to release payload. (**b**) Schematic representation of maytansinoid ADC linker highlighting the role of alpha-methyl groups (R1 to R4). (**c**) Linker structure of SAR-3419 containing the DM4 payload and the SPDB linker.

Disulfide-containing linkers undergo nucleophilic attack by thiols to release the active payload (Figure 44a). Although the most abundant thiol in blood plasma is the reduced form of human serum albumin (HSA), its reactivity towards large molecules is poor [66]. The cytosol also contains high levels of gluthatione (GSH), a thiol-containing tripeptide that reacts readily as S-nucleophile. The difference between GSH concentration in blood (micromolar range) and cytoplasm (milimolar range) and the oxidative stress caused by cancer cells contribute to the preferential intracellular release of the drug. Disulfide-containing linkers are mainly associated with the maytansinoid payload class. The reactivity of the disulfide bond can be modulated by steric hindrance: alpha methyl (poly)substitutions (R1-R4 in Figure 44b) significantly affect the rate of reduction and the resistance to thiol-disulfide exchange [67]. For example, the linker structure of clinical candidate SAR-3419 was fine-tuned for optimal antitumoral activity selecting SPDB-DM4, which displays a gem-dimethyl substitution on the payload side (Figure 44c) [68].

**Figure 45:**
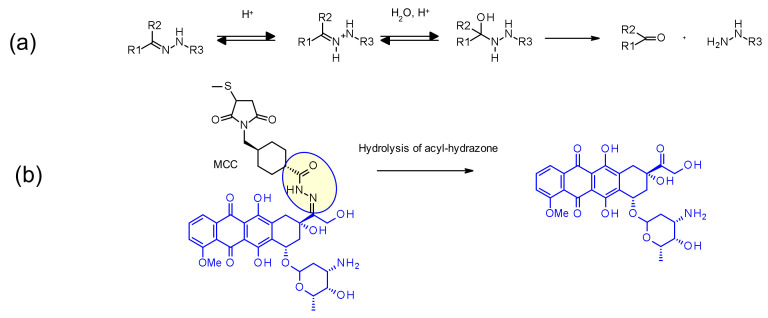
(**a**) Hydrolysis of hydrazone in acidic conditions (**b**) Cleavage of acyl hydrazone linker present in IMMU-110, releasing doxorubicin.

Hydrazone linkers display a pH-dependent stability, being stable at neutral pH (blood stream) and being hydrolized in acidic media (pH < 6 for endosomes then pH < 5 for lysosome) to form the corresponding ketone and hydrazine (Figure 45a). This approach has been applied with success in IMMU-110, containing a cleavable acyl hydrazone linker formed in the reaction of the hydrazide of 4-maleimidomethyl cyclohexane-1-carboxylate (MCC) and the keto group present in doxorubicin (Figure 45b) [69]. Hydrazone linkers are also frequently associated with the calicheamicin payload family: in this context, the release is triggered by a two-step’s activation process: first the acid-sensitive hydrazone is hydrolyzed and in the second step the disulfide-bond is reduced by GSH, allowing cyclization of the sulfhydryl intermediate [70] (Figure 28). Even if this linker reached the market twice (by the earliest approved ADC Mylotarg^®^ [71] and then Besponsa^®^ [72]), hydrazine-based linkers have demonstrated discrepancies between plasma and buffer stability. They are less stable in plasma than expected and less attractive than other cleavable linkers by direct comparison [73].

#### 3.2.2. Enzymatic Cleavage

To limit release of the payload before internalization, thus preventing or minimizing degradation outside the target cell, the proteome of the lysosome became a logical place to search for enzymes capable of ADC degradation and present in high concentration [74].

##### Cathepsin-B

**Figure 46:**
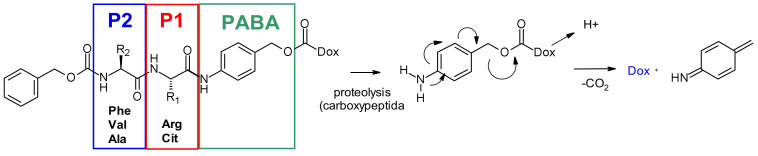
Release mechanism from the dipeptide-PABC-doxorubicin conjugate. Doxorubicin, linked by its primary amine, is not present for clarity.

Cathepsin B, a cysteine protease presents in late endosome and lysosome compartments in mammals, is also overexpressed in many cancer cells. Initially, cleavable dipeptides were explored as cathepsin B substrates for doxorubicin prodrugs [75]. This work has established the basis of SAR of the dipeptide moiety (Figure 46): A hydrophilic residue is required at position P1 (citrulline or arginine), whereas a lipophilic residue at position P2 enhances plasma stability (phenylalanine, valine or alanine). In addition, a self-immolative spacer was introduced to facilitate enzyme access, limiting the steric hindrance of the payload: para-aminobenzyl carbamate (PABA) undergoes spontaneous 1,6-elimination in acidic media releasing carbon dioxide, para aza-quinone methide and doxorubicin. This initial finding was then transferred with success from prodrugs to the ADC field, using BR96-antibody, demonstrating antigen-driven cellular activity with Val-Cit and Phe-Lys dipeptide linkers [76].

The Val-Cit dipeptide is the most common used cleavable linker in ADCs (with an impressive prevalence of 25 examples in the clinic), likely because of its overall good plasma stability, release behavior, and chemical tractability (contrary to Arg or Lys-containing dipeptides, no need for protecting groups). Two approved ADCs (Adcetris^®^ and Polivy^®^) share the same linker construct mc-VC-PABC, containing a maleimidocapryl spacer, the standard Val-Cit dipeptide sequence as cathepsin substrate, and a PABC self-immolative spacer (Figure 2).

**Figure 47:**
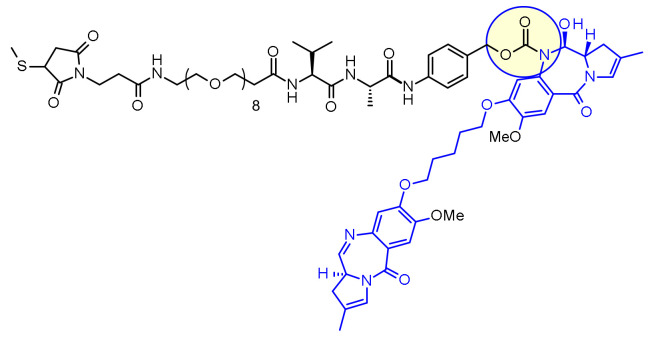
Chemical structure of Loncastuximab tesirine.

The dipeptide Val-Ala has also been also extensively used, with seven examples in clinic, the most advanced being Loncastuximab tesirine, displaying a pegylated spacer to balance the lipophilicity of the payload SG3199, belonging to the PBD dimer family (Figure 47). An elegant side by side comparison between Val-Cit and Val-Ala linkers in the context of anti-CD30 and anti-CD70 ADC vectorizing Doxorubicin derivatives demonstrated that achieving a high DAR with Val-Cit is difficult due to precipitation and aggregation [77]. In contrast, the Val-Ala linker allowed DAR up to 7.4 with limited aggregation (<10%). According to the authors, the (non-obvious) less hydrophobic behavior of Val-Ala compared to Val-Cit explains why this linker is superior in the context of lipophilic payloads, such as PBD-dimers (the seven clinical candidates featuring Val-Ala linkers are all releasing PBD). In addition, the use of such a dipeptidic linker, containing two amino acids with lipophilic residues goes against the initial findings that cathepsins cleaved sequences of a hydrophilic residue and a lipophilic residue.

Several publications compare Val-Cit and Val-Ala dipeptide constructs with MMAE as payload. In case of the non-internalizing F16 antibody, both Val-Cit and Val-Ala linkers conjugated to engineered cysteine exhibit comparable profiles and better performance than strict Val-Lys and Val-Arg analogues [78]. In the context of an anti-Her2 ADC using stochastic Cysteine conjugation, Val-Ala displayed less aggregation for high-DAR constructs compared to Val-Cit. On the other hand, both linkers displayed comparable buffer stability, Cathepsin B release efficiency, cellular activity, and histopathology profile [79]. Alternatively, using rebridging format, the Val-Cit linker leads to higher DAR distribution than Val-Ala with a hydrophobic spacer (caproyl) [80]. If the spacer is modified, incorporating a linear PEG12 moeity, the difference in terms of DAR distribution becomes non-significant. So, the addition of solubilizing PEG units in the linker is enough to overcome the intrinsic difference of physicochemical properties. In addition, different cellular activity was observed for Val-Cit and Val-Ala-containing ADCs (regardless of the presence or absence of PEG units).

**Figure 48:**
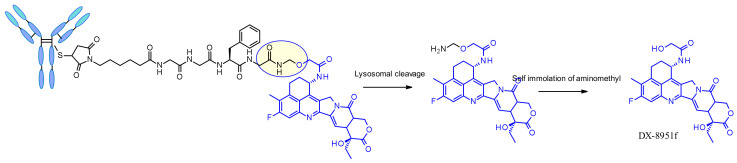
Chemical structure of trastuzumab deruxtecan.

The tetrapeptide Gly-Gly-Phe-Gly displays all the characteristics of a stable and potent cleavable ADC linker and was brought to the market successfully with trastuzumab deruxtecan, Enhertu^®^ (Figure 48) [81]. Researchers from Daiichi Sankyo have designed a plasma stable ADC with a DAR of 7.7 and demonstrated that DS-8201a undergoes protease degradation in the lysosome releasing DX-8951f, a potent Topoisomerase I inhibitor derived from exatecan [82]. As the linker does not contain solubilizers, reaching such high DAR is remarkable as it contradicts the widely established principle that high DAR conjugates may have poor pharmacokinetic profiles. The self-immolative spacer used here is the simple and compact hemiaminal, instead of the PABC used with Val-Cit linker.

##### Phosphatase and Pyrophosphatase

**Figure 49:**
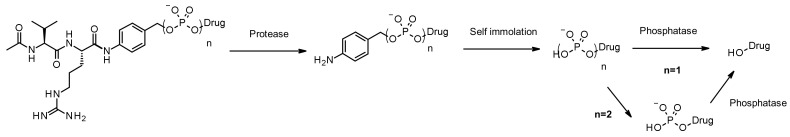
Schematic structure and postulated release mechanism of phosphate (*n* = 1) and pyrophosphate (*n* = 2) linkers. Conjugation type to antibody and payload (budesonide) not represented for clarity. Budesonide is linked by its primary alcohol.

Like cathepsins, pyrophosphatases and phosphatases are hydrolases exhibiting selective expression in the lysosome. In 2016, researchers at Merck designed phosphate and pyrophosphate-containing linkers coupled with the well-established, cathepsin B sensitive, Val-Cit-PABA moiety aiming to deliver glucocorticoïds (Figure 49): the phosphate/pyrophosphate moiety is incorporated between the self-immolative spacer PABA and the payload (budesonide) [83]. After internalization, the payload may be released via an original sequence involving cathepsin B, self-immolation and phosphatase (for *n* = 1). In the case of pyrophosphate esters (*n* = 2), another step involving pyrophosphatase is likely required. The authors demonstrated that efficient payload release is achieved by induction with lysosomal extracts. The advantage of using such hydrophilic and permanently charged moieties is the solubility, enabling not only bioconjugation of the chemical precursor incorporating a greasy strained cycloalkyne and a lipophilic glucocorticoid derivative, but also facilitating ADC purification, leading to less than 0.10% of residual linker in the ADC. Both phosphate and pyrophosphate-containing ADC are active in vitro.

**Figure 50:**
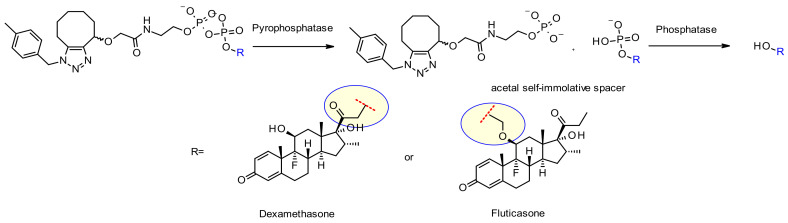
Chemical structure and release mechanism of pyrophosphate diester linkers.

The same group of researchers at Merck also developed a unique pyrophosphatase-based linker applied in anti-CD70 ADCs releasing hydroxyl-containing payloads dexamethasone and fluticasone propionate (Figure 50) [84]. The scope and limitations of this new phosphate diester linker was established: when the linker contains only a monophosphate moiety, the release in suboptimal. 

In addition, the nature of the hydroxyl-containing attachment point is crucial for efficient release. Primary alcohol of dexamethasone works well, whereas the more hindered fluticasone secondary alcohol requires an acetal spacer to show acceptable release. Both ADCs exhibit good in vitro mouse and human plasma stability over seven days and strong activity against CD70+ cell lines.

##### β-Glucuronidases

**Figure 51:**
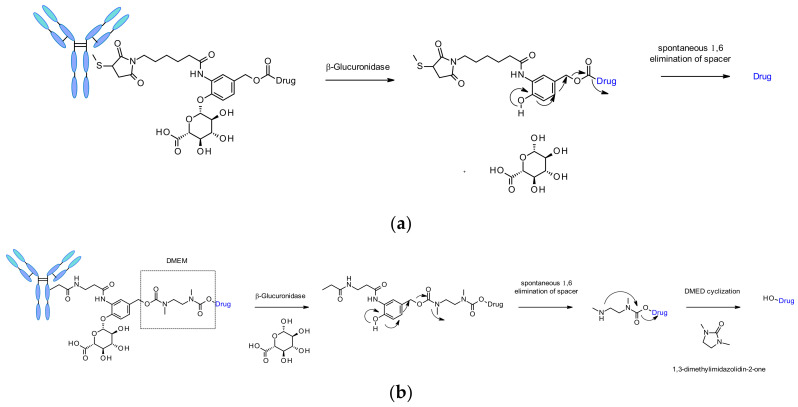
(**a**) Chemical structure of β-glucuronic linker and corresponding release mechanism. Drugs contains primary (doxorubicin) or secondary amine (MMAE, MMAF) (**b**) Chemical structure and release mechanism of DMED-containing β-Glucuronidase cleavable linker. Drug structures are omitted for clarity.

β-Glucuronidases are a class of glycosidase enzymes, which catalyze the hydrolysis of β-glucuronic residues. Its abundance in lysosomes and tumor interstitium associated with the hydrophilicity of its substrates are the reasons of interest for the design of safe and efficient cleavable linkers for ADC. A seminal work was published in 2006 by researchers from Seattle Genetics describing anti-CD70 ADCs releasing amine containing MMAE, MMAF and doxorubicin payloads (Figure 51a) [85]. This original linker contains a β-glucuronic moiety attached to a self-immolative spacer. The link towards the antibody is located optimally in ortho position to the cleaved bond. Such linkers demonstrated low level of aggregation, high plasma stability, a cleavage process validated in vitro upon incubation with the corresponding enzyme, and robust in vivo efficacy. This linker has also been applied to other amine-containing payloads such as CBI minor groove binder [86], camptothesin analogues [87] and hydroxyl-containing molecules SN38, duocarmycin and psymberin via an additional dimethyl ethylene diamine (DMED) self-immolative spacer (Figure 51b) [88]. The release sequence starts by the hydrolysis of the β-glucuronic acid followed by self-immolation. An additional cyclization of DMED occurs spontaneously to form 1,3-dimethylimidazolin-2-one and eventually release the hydroxyl-containing drug.

Due to the hydrophilicity of the linker, this technology allows easier preparation of ADCs with DAR 8 compared to cathepsin-sensitive linkers [89].

##### β-Galactosidase

**Figure 52:**
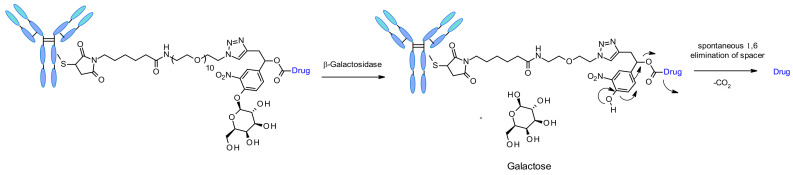
Chemical structure of β-galactosidase linker and mechanism of release.

Recently the use of β-galactosidase cleavable linkers for ADCs was reported incorporating a PEG10 spacer (Figure 52) [90]. The spacer was substituted by a nitro group in order to increase the rate of self-immolation [91]. By analogy of β-glucuronidase linkers, the cleavage mechanism involves the hydrolysis of the β-galactosidase moiety, which confers hydrophilicity to the chemical precursor. Another advantage is that the β-galactosidase enzyme is present only in the lysosome, whereas β-glucuronidase is expressed in lysosomes and also in the microenvironment of solid tumors [92]. The authors demonstrated that, in the context of anti-HER2 ADCs releasing MMAE, β-galactosidase-containing ADCs are more potent than the gold standard Traztuzumab emtansine (T-DM1) both in vitro and in vivo.

##### Sulfatase

**Figure 53:**
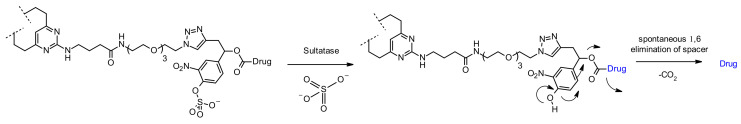
Chemical structure of sulfatase-cleaved linker and the corresponding release mechanism. Both drug structure and antibody omitted for clarity (rebridging conjugation).

Very recently, sulfatase-cleaved linkers emerged [93]. As glycosidases and (pyro)phosphatase-based linkers, they display good hydrophilicity due to the nature of the substrate of interest (permanently-charged sulfate) while being mainly expressed in the lysosome. In addition, sulfatases are overexpressed in several cancer types, suggesting a potential additional selectivity [94]. Authors have rationally designed linkers, assessed cleavage rates in vitro before the use with MMAE as payload and anti-Her2 antibody. Compared to classical cleavable Val-Cit and Val-Ala linkers, sulfatase linkers show similar cellular potencies against Her2+ cell lines (Figure 53). In parallel to the academic comparison with standard linkers, sulfatase linkers have also recently attracted industrial attention for potential development [95].

## 4. Bioconjugation

The design of clinically successful ADCs is defined not only by payload potency and its attachment point, linker stability and efficient drug release, but also by the choice of antibody (mAb) and bioconjugation technology. For the past 10 years, all ADCs approved by the FDA were in the form of a heterogeneous mixture of ADCs bearing varying numbers of drugs attached at different positions on the mAb. Though the conjugation site has significant impact on ADC stability and its pharmacokinetics-pharmacodynamics profile. High drug loading often leads to rapid plasma clearance and ADCs with low DAR (drug-to-antibody ratio) demonstrate weak activity [3]. The presence of naked mAb in the mixture presents a potent competitive inhibitor in these formulations. Thus, for the past decade, a large panel of new conjugation strategies has been developed aiming to control the position and the number of drugs, while maintaining structural integrity and homogeneity. In this chapter, we review the recent progress in site-specific conjugation on native and engineered antibodies designed to produce homogeneous ADCs.

### 4.1. Chemistry Based Site-Specific Modification of Native Antibodies

The structure of native mAbs offers multiple possibilities for bioconjugation. Thus, the chemistry-based, site-specific conjugation of native (non-engineered) antibodies presents several advantages. It allows to avoid the complexity of determining suitable mutation sites and potential challenges for scaling up and optimization of the cell culture [96]. Depending on the antibody sequence, endogenous amino acids such as lysine, histidine, tyrosine and cysteines engaged in interchain disulphide bridges, are attractive attachment sites. All ADCs approved by the FDA until 2021 exploit some of these endogenous amino acids for conjugation. However, the antibody scaffold also contains glycan incorporated into the Fc region resulting from a common post-translational modification during mAb production. Several groups have reported new strategies of glycoengineering which appear to be an interesting alternative for bioconjugation.

#### 4.1.1. Conjugation to Endogenous Amino Acids

One of the most common conjugation methods exploits the antibody’s lysine residues, where the nucleophilic NH_2_-group of the amino acid reacts with an electrophilic *N*-hydroxysuccinimide (NHS) function on the liker-payload. Despite the simplicity of the reaction, the high abundance of accessible lysine residues leads to the formation of a heterogeneous mixture of numerous ADC species in stochastic distribution. The DAR is controlled by the drug/mAb stoichiometric ratio. This methodology was widely used to produce clinically approved ADCs such as Besponsa, Mylotarg, and Kadcyla [97,98].

**Figure 54:**
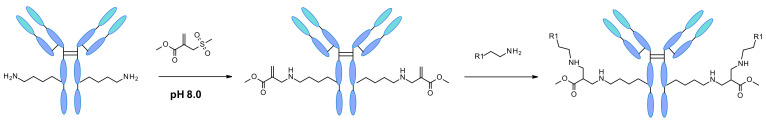
Sulfonyl acrylate reagent reacting with lysine residues of native mAb.

Recently site and residue-specific modifications on lysine by chemical reagents were also reported [99]. Following computer-assisted design sulfonyl acrylate was used as reagent for the modification of a single lysine residue on native protein sequences (Figure 54). The regioselectivity of the reaction was due to the combination of the designed sulfonyl acrylates and the unique local microenvironment surrounding each lysine. Matos et al. predicted computationally that the lysine with the lowest pKa was prone to react preferentially at slightly basic pH in a site-specific manner. The conjugation was observed even in the presence of other nucleophilic residues such as cysteine. This technology was applied on five different proteins and trastuzumab, demonstrating quantitative and irreversible modification with retained native secondary structure and protein functionality after conjugation.

**Figure 55:**
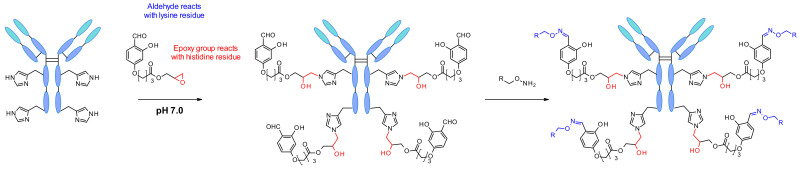
Linchpin directed conjugation on histidine residues performed by multitasking group reagent.

In 2018, Rai et al. reported another site-specific modification by exploiting a reversible intermolecular reaction using “chemical linchpins” [100]. The reagent carries multiple functional groups, which form imine moieties reversibly on all accessible lysine residues (Figure 55). Then the linchpins react with proximal histidine residues through the epoxide present in the reagent. Thus, the linchpin detaches from the lysins under physiological conditions and the aldehyde is regenerated, enabling the labeling of the antibody by oxime conjugation.

**Figure 56:**
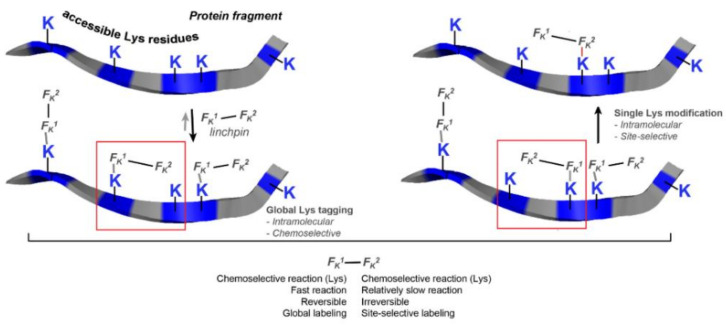
Hypothetic mechanism of linchpin technology for site-specific conjugation on lysine residues based on F_k_^1^-spacer-F_k_^2^ reagent.

This linchpin directed modification technology was later developed into single lysine residue labelling with uncompromised selectivity even in the presence of *N*-terminal amine [101]. The success of the methodology relies on F_k_^1^-spacer-F_k_^2^ reagent (Figure 56). F_k_^1^ functional group reacts with the accessible lysine trough reversible reaction and regulates the microenvironment of F_k_^2^ near to a proximal Lys moiety. Then the conjugation though amide bond is performed between F_k_^2^ at Lys residues (K169 and K395). The design of the spacer regulates the site of conjugation. This methodology was applied successfully for the synthesis of ADC (trastuzumab-emtansine) that demonstrated comparable cellular activity to FDA approved Kadcyla. The linchpin methodology operates with multiple proteins possessing a wide range of structural complexity.

**Figure 57:**
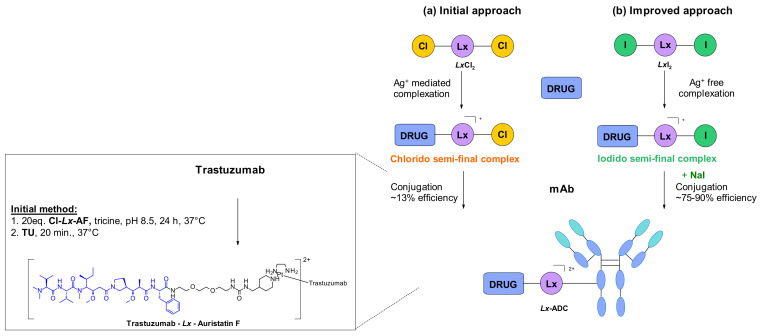
Site-specific conjugation approach on histidine residues by cationic metal-organic Pt^II^-based linker: (**a**) initial approach generating chlorido semi-final complex. (**b**) Improved approach through iodido semi-final complex.

A different conjugation strategy efficiently targeting histidine residues on native antibodies was recently reported by Merlul et al. [102]. They introduced an original cationic organometallic Pt(II)-based linker, [ethylenediamineplatinum(II)]^2+^ denoted Lx (Figure 57). This technology is based on a two steps complexation and conjugation sequence [103]. N-heterocyclic ligands such as piperidine are coordinated to Lx establishing a precursor complex. The stable intermediate contains the payload and one chloride leaving group on the ligand (Figure 57a). This complex contains positively charged Pt(II) center, which improves the water solubility of the linker-payload conjugate and minimizes antibody aggregation. The approach was also extended to the analogous iodido complex, which was beneficial for the Lx conjugation (Figure 57b) [104]. In a recent report, the use of NaI was shown to improves significantly the conjugation yield and selectivity of the technology. The exchange of the leaving chloride ligand on Cl-Lx-drug complexes to iodide generates the more reactive I-Lx-drug species, resulting in higher conjugation yield. Then the high affinity of Pt(II) to the N-donor, such as the imidazole of histidine residues, drives the conjugation step. This technology was applied to larger ADC production for toxicological studies and first-in-human trials.

#### 4.1.2. Disulphide Rebridging Strategies

**Figure 58:**
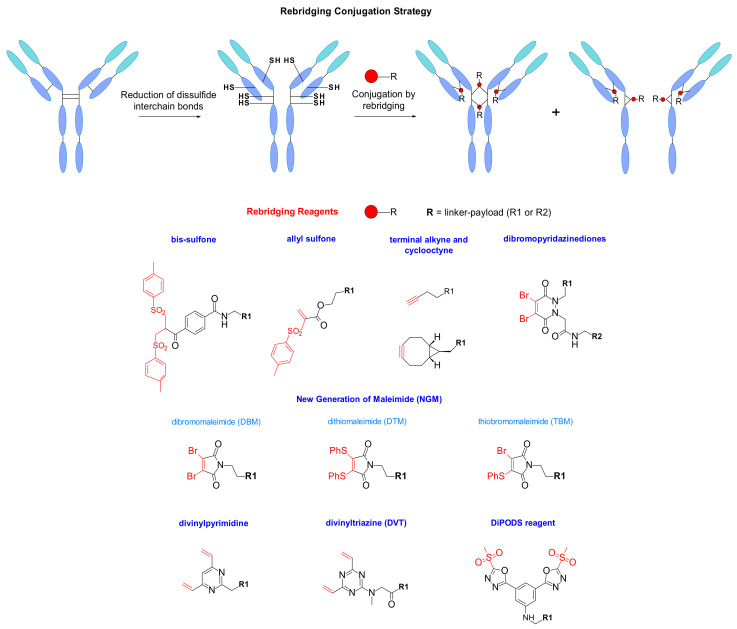
Rebridging conjugation strategy for modification of native antibodies using rebridging reagents.

IgG1 antibodies contain four interchain disulphide bonds, two connecting the light and heavy chains, and two located in the hinge region bridging the two heavy chains, all together maintaining the integrity of the mAb [105]. Another classical bioconjugation route explores these cysteines as payload-linker attachment points to the mAb. The reduction of the four disulphide bridges normally generates eight thiol groups that are able to react with maleimide functionalized linkers resulting in DAR 8. An example of DAR 8 ADC on chimeric anti-CD30 mAb carrying MMAE was reported by Doronina and coworkers [73]. This way of payload loading is controlled better compared to the classical lysine conjugation. However, it has been reported that higher drug loading increases the risk of aggregation leading to high plasma clearance rate, and decreased in vivo efficacy [106,107]. A new site-specific rebridging conjugation strategy was reported in 2014 by Badescu and al, who were the first to demonstrate that a new bis-sulfone reagent was capable of alkylating both thiol groups derived from a reduced disulphide bond in antibodies and antibody fragments, with minimal impact on antigen binding (Figure 58) [108]. Later, a new water-soluble allyl sulfone was described by Wang and al, where the reagent had improved reactivity without in situ activation. It demonstrated high stability, high water-solubility, and site-specificity. The reagent was mostly used on peptides and proteins, and the combination of three different functionalities at a single site offered access to different customized bioconjugates [109].

The rebridging technology was also described using thiol-yne bioconjugation with terminal alkyne and cyclooctyne (Figure 58). After reduction of the disulfide bond, the crosslinking was regenerated through photo-mediated insertion of the functionalized two carbon linker. This methodology was applied to different peptides, but also to antibody fragment Fab showing promising conversion [110].

Additionally, the strategy was completed by the work of Schumacher and al, who reported a new generation of maleimides such as dibromo- (DBM) and dithiomaleimide (DTM) for site-specific conjugation [111] (Figure 58). These maleimide analogues contain good leaving groups at position 3 and 4 resulting in fast, efficient, and high yielding conjugation. They lead to the substitution with the two nucleophilic thiol groups and enable better stability compared to classic maleimides, which are prone to retro-Michael reaction. Recently it was reported the synthesis of hybrid thiobromomaleimide (TBM) combining the properties of dibromo- and dithiomaleimide [112]. This TBM reagent conjugates faster and demonstrates higher percentage of DAR 4 probably due to the bromide that reduces steric hindrance and represents a better living group than the thiophenolate.

In 2015, Chudasama et al. introduced a new class of rebridging reagents, the dibromopyridazinediones [113] (Figure 58). They demonstrated its efficient insertion into disulfide bonds and the resulting constructs displayed exceptional stability to hydrolysis, even at high temperatures. However, heterogeneity was observed as the temperature on the reduction step increases [114]. The structure of this reagent also allows the selective introduction of distinct functionalities.

Divinylpyrimidine was also found to be an efficient rebridging reagent leading to serum stable antibody conjugates with consistent DAR 4 [115,116]. Spring et al. studied the usefulness of a vinylheteroaryl scaffold for cysteine rebridging (Figure 58). They suggested that the replacement of the pyridine by a pyrimidine should enhance the crosslinking efficiency through making the heteroaryl ring a better electron acceptor. Their work was extended to divinyltriazine where the rebridging demonstrated increased efficiency at high temperature [117]. It needs to be pointed out that the presence of the undesired ‘half-antibody’ remains an issue in the development of disulphide rebridging linkers. However, the DVT technology demonstrates high efficiency using a stochiometric amount of reagent, which represents an advantage for the development of this conjugation.

To circumvent the drawback of in vivo instability associated with classical maleimide linking, Barbas et al. studied the methylsulfonyl-phenyloxadiazole reagent, which demonstrates specific reactivity for cysteine (Figure 58). They reported a superior stability compared to cysteine maleimide conjugates in human plasma [118,119]. Inspired by this report, Zeglis designed the reagent DiPODS for site-specific, irreversible bioconjugation on native antibodies fragment Fab [120,121,122]. This reagent contains two oxadiazolyl methyl sulfone moieties connected by a phenyl group. These DiPODS form a covalent bond with two thiolate groups in a rebridging manner. Computational analysis also confirmed the superior thermodynamic stability of the DiPODs conjugates compared to monovalent PODS, mono- and bivalent maleimides. The conjugates synthesized in this manner had a superior in vitro stability and in vivo performance compared to a maleimide-based probe. 

It has been noted that the rebridging process can generate fragmentation due to the formation of intrachain bridges between hinge region cysteines inside the heavy chain. The fragmentation resulting in “half-antibody” species yields a mixture of two antibody conjugates. In this context, multiple reagents and conditions have been designed to generate more homogeneous ADCs. Up to date there is no study suggested that the presence of “half-antibody” species has a negative effect on the antibody’s profile [123]. Therefore, the rebridging strategy appears to be a promising bioconjugation technique on native mAbs successfully retaining the key properties of the native mAb, and allowing to generate DAR 4 species. 

#### 4.1.3. Conjugation to Glycan

As the human IgG is a glycoprotein, it contains an *N*-glycan at position N297 in each heavy chain on the CH2 domain of the Fc fragment. This glycosylation could be exploited as an attachment point for the linker-payload. The glycan’s distant localization from the Fab region decreases the risk of compromising the antibody’s antigen binding ability after conjugation. Moreover, their different chemical composition compared to the antibody’s peptide chain, allows site-specific modification, making them a suitable conjugation site.

Glycan bioconjugations can be distinguished based on the technique used to target the carbohydrate: glycan metabolic engineering, glycotransferase treatment followed by glycan oxidation, endoglycosidase and transferase treatment coupled with keto- or azide tagging.

**Figure 59:**
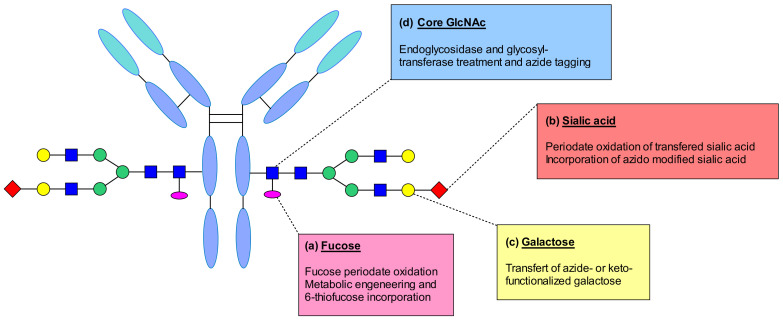
(**a**) Fucose modification by oxidation with periodate or by metabolic engineering of thiolated analogue. (**b**) Enzymatic addition of terminal sialic acids followed by periodate oxidation or incorporation of azido modified sialic acid. (**c**) Incorporation of azido- or keto- functionalized galactose. (**d**) Endoglycosidase homogenizing the glycan structure and incorporation of an azide anchor enabling copper-free cycloaddition.

Neri et al. reported a site-specific modification on the fuctose attached to the GlcNAc unit at the *N*-glycosylation site of IgG antibody (Figure 59a). This sugar contains a cis-diol moiety suitable for selective oxidation. They used sodium metaperiodate to oxidize the fuctose residue generating an aldehyde moiety able to react with hydrazine containing linkers. Thus, the antibody was linked to the drug by hydrazone linkage [124].

Fucose derivative by metabolic incorporation into the antibody was described by Senter and his coworkers [125] (Figure 59a). They reported the incorporation of 6-thiofucose by supplementing the cell culture medium with the thiolated analogue. They suggested that the substitution was performed by hijacking the fucosylation pathway, which resulted in the introduction of a chemical handle for site-specific conjugation. This approach significantly decreases the level of heterogeneity compared to classical cysteine conjugations and generates conjugates with more predictable pharmacokinetic and pharmacodynamic properties.

Recombinant IgG contains rarely sialic acids. However, it has been demonstrated that using galactosyl and sialyltransferases, it is possible to enzymatically remodel glycans. Zou et al. demonstrated the enzymatic addition of galactose to obtain a G2 glycan, followed by the addition of terminal sialic acids (Figure 59b) [126]. This modification enabled the generation of an aldehyde group through periodate oxidation and the functionalization of the antibody using a hydroxylamine functionalized linke-payolad. The obtained conjugates demonstrated high target selectivity in vitro, and good in vivo anti-tumor activity. It was suggested that periodate could also oxidize sensitive amino acids such as methionine, which could affect FcRn binding. A different strategy was reported by Li et al. exploiting the tolerance of glycosyltransferases to chemically modify the sugar substrates [127]. Their study described the transfer of galactose and incorporation of azido-modified sialic acid in a two-step enzymatic process. The anti-CD22 mAb was treated with galactosyltransferase and UPD-Gal in order to create the maximum number of acceptor sites for a sialyltransferase. In the second step, a sialic acid derivative with azide anchor was incorporated. Then the mAb underwent copper-free click-chemistry with appropriately functionalized linker-payload generating an ADC with a DAR of 4.3.

In addition to these conjugation strategies, the galactose residue was also explored as a modification site. Multiple studies reported the removal of galactose using beta-1,4 galactosidase, followed by the incorporation of galactose derivatives having biorthogonal handles (Figure 59c) [128,129,130]. By using a mutant β-1,4-galactosyltransferase, a galactose bearing a ketone or azide functional group was introduced, which opened the way for efficient conjugation by biorthogonal transformations. These techniques were exploited for imaging and anticancer applications.

The discovery of endoglycosidases EndoS and EndoS2 from the human pathogen Streptococcus pyogenes, which are able to hydrolyze *N*-linked glycans of human IgG, enabled the targeting of the IgG GlcNAc residue for bioconjugation (Figure 59d) [131]. This approach helps to homogenize the glycan structure of the mAb and it is applicable to any IgG isotype despite the glycosylation profile. A new technology called GlycoConnect was reported to perform a highly controlled attachment of any payload to the antibody’s glycan at asparagine-297 [132]. First, an IgG-specific endoglycosidase performs the enzymatic remodeling to expose the core GlcNAc of the native antibody glycan. Following the glycan cleavage, the engineered galactosyl transferase GalT in combination with UDP-GalNAz incorporates an azide anchor, where biocompatible, copper-free, strain-promoted azide–alkyne chemistry reaction could be performed subsequently. This approach was applied to trastuzumab and maytansine to produce GlycoConnect ADCs that demonstrated favorable in vitro potency and in vivo efficacy.

Glycoengineering is an attractive bioconjugation technique on native antibodies, although the high variability of the glycan structure can be an obstacle for the conjugation of a payload. The ratio of isoforms depends on the antibody isotype and the mammalian expression system used to produce the mAb. The efficient remodeling of the antibody’s glycans requires a detailed knowledge of their compositions. The different glycoengineering strategies demonstrated promising results, and their efficiency is getting close to the site-specific bioconjugation of engineered antibodies.

### 4.2. Site-Specific Bioconjugation of Engineered Antibodies

The advances in the fields of bioorthogonal chemistry and protein engineering help generating more homogeneous ADCs. Despite the considerable choice of available attachment methodologies on native mAbs, the site-specific bioconjugation on engineered antibodies carries the advantages of controlling more efficiently the DAR, and the reaction site avoiding altering the antigen binding affinity. In this way, the incorporation of natural or unnatural amino acids at certain positions gives homogeneous products with excellent pharmacokinetic and pharmacodynamic profiles.

#### 4.2.1. Enzymatic Approaches

The attachment of the payload can be achieved in a very selective manner by using genetically encoded amino acid tags inserted in the antibody sequence. These tags are specifically chosen to be recognized by an enzyme, such as formylglycine-generating enzyme (FGE), microbial transglutaminase (MTG), sortase, or tyrosinase, capable to perform site-specific conjugation.

**Figure 60:**
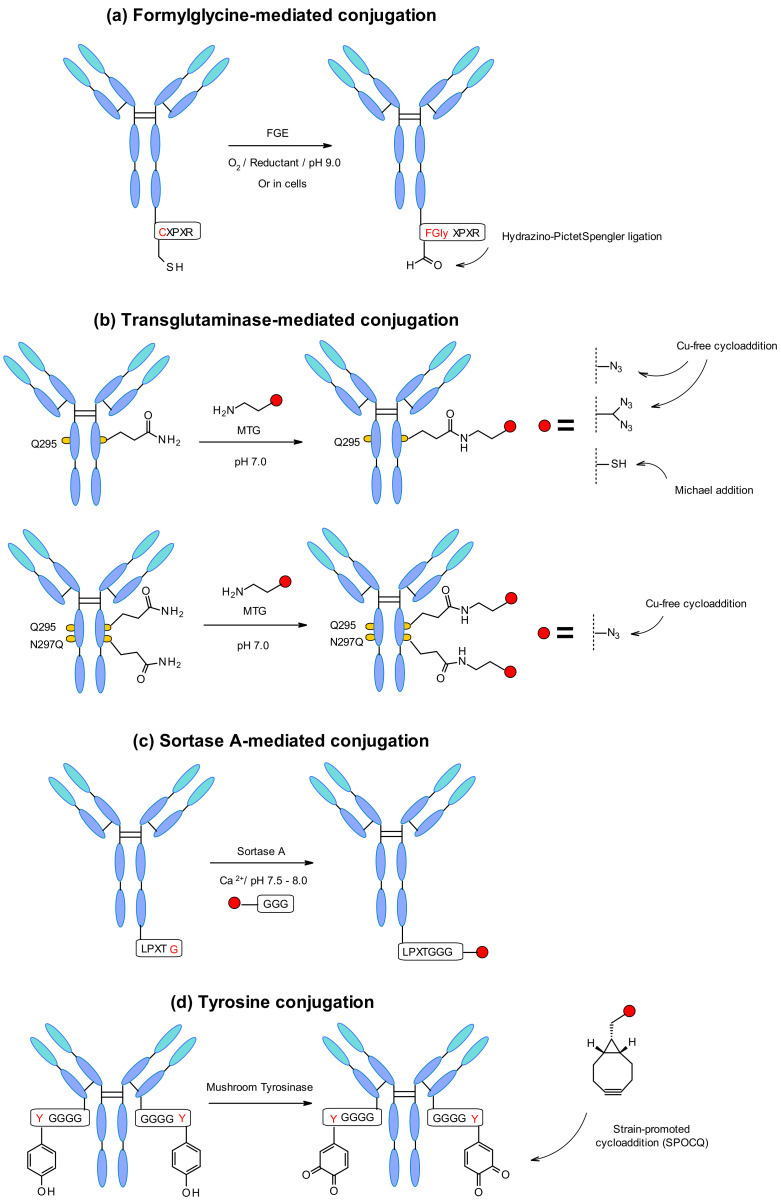
(**a**) Cysteine residue in pentapeptide Cys-X-Pro-X-Arg oxidized to formylglycine by FGE generating bioothogonal handle. (**b**) Microbial transglutaminase (MTGase) strategy on glutamine at position 295 and improved approach by incorporation of additional mutation N297Q. (**c**) Sortase-mediated conjugation exploiting glycine-functionalised payloads. (**d**) Tyrosine oxidation by mushroom tyrosinase generating biorthogonal handle for strain-promoted cycloaddition. For clarity, the reaction (**a**) and (**c**) is only depicted in one heavy chain.

Aaron et al. explored a novel site-specific conjugation chemistry that takes advantage of an aldehyde-tagged protein [133]. The technology exploits a genetically encoded pentapeptide sequence (Cys-X-Pro-X-Arg) where the cysteine residue is recognized by FGE and oxidized co-translationally to formylglycine during protein expression in cells (Figure 60a). Thus, the engineered antibody reacts selectively through the hydrazino-Pictet–Spengler ligation with aldehyde-specific warheads.

The microbial transglutaminase (MTGase) strategy is also frequently exploited to site-specifically conjugate different payloads. The MTGase enzyme catalyzes the formation of a peptide bond between the glutamine side chain at position 295 of the deglycosylated antibody and the primary amine of the substrate (Figure 60b). In contrast to other enzymatic strategies, MTG is a flexible technology that does not require a peptide donor in order to perform the coupling. There are no structural limitations about the acyl acceptor as long as it contains a primary amine [134]. It might contain, for example, a chemical handle for thiol-maleimide or strain-promoted azide–alkyne cycloaddition (SPAAC) in order to incorporate the linker-payload [135].

The glutamine residue is naturally present in the Fc region of each heavy chain of the mAb. After deglycosylation of position 295, glutamine can conjugate through MTGase-mediated transpeptidation of the linker-payload affording homogeneous ADCs with DAR2. In order to increase the efficiency, branched linkers could be attached to the glutamine residue leading to doubling the DAR [136] (Figure 60b). The DAR could also be increased by mutation of asparagine 297 to a glutamine at this position. This strategy was developed by Innate Pharma using an aglycosylated variant of the anti-CD30 antibody with N297Q mutation [137]. The in vitro and in vivo stability demonstrated by the homogenous conjugates was comparable to the pharmacokinetics, safety and efficacy profile of Adcetris. Recently MTG mutants have being designed to enable the efficient, direct, site-specific incorporation of drug-linkers, and reactive handles on different native and glycosylated mAbs inside the antibody structure, without the need to insert an additional handle [138].

It was also demonstrated by Spidel et al. that the native lysine 447 residue can act as a highly efficient acyl acceptor when using microbial transglutaminase [139]. As the residue is cleaved by carboxypeptidase B in the host production cell, the cleavage was blocked by the addition of a C-terminal amino acid at position 448 allowing access to the lysine residue.

A further enzymatic conjugation approach, based on the *S. aureus* sortase A-mediated transpeptidation, was developed by NBE Therapeutics. Their strategy exploits the enzyme sortase A (Srt A) that cleaves the amide bond between threonine and glycine residues in the LPXTG (X = any amino acid) pentapeptide motif (Figure 60c). Then, it catalyzes the linking of glycine-derived payloads to the newly generated C-terminus, generating a peptide bond at physiological temperature and pH [140]. This approach was applied to different antibodies such as anti-CD30 and anti-Her2 with penta-glycine tagged linkers containing maytansine and MMAE [141]. Both ADCs display in vitro cell killing activity comparable to the classic conjugates. The enzymatically generated trastuzumab-maytansine tested in vivo led to complete regression similar to Kadcyla. In another example anthracycline-based antibody–drug conjugates were generated by sortase-mediated conjugation [142]. The ADCs were synthesized using a highly potent anthracycline toxin derivative PNU-159682, and a non-cleavable peptide linker conjugated to the two previously described mAbs. Interestingly, the conjugation efficiency through this technology was even higher than for Adcetris and Kadcyla analogues. Consequently, the conjugation efficiency depends on the nature of the linker-payload structure. In addition, Stefan et al. demonstrated that the obtained PNU-159682 ADC had high in vitro and in vivo stability and exhibited potencies exceeding those ADCs that contained tubulin-targeting payloads.

Another emerging new methodology is the site-specific antibody labelling through a tyrosine tag genetically fused to the C-terminus of the mAb light chains (Figure 60d) [143]. Given the inaccessibility of the native tyrosine, Bruins and coworkers used an engineered tetra-glycyltyrosine residue as a tag. It offered an easy reachable site for coupling, where mushroom tyrosinase oxidized the tyrosine to 1,2-quinone allowing a strain-promoted cycloaddition with various bicyclo[6.1.0]nonyne (BCN) derivatives. This approach worked efficiently with an MMAE containing BCN linker showing a rapid and selective conjugation under physiological conditions.

There are accumulating evidences that the enzymatic methods enable easily achieved amino acid modifications. They allow directly the conjugation of the linker-payload to the mAb or by introducing a biorthogonal handle first, which can be later functionalized with the drug. The high specificity of these methods guarantees the site-specificity of the conjugation and the mild reaction conditions help to preserve protein integrity.

#### 4.2.2. Cysteine Engineering: Thiomab Technology

As described in Section 4.1.2, stochastic cysteine conjugation and rebridging are techniques that exploit the naturally present cysteine residues inside the antibody structure. Both methods are adopted on native mAbs. However, the heterogeneity of the stochastic cysteine approach, and the mAb fragmentation in the rebridging strategy need to be considered in ADC synthesis, especially when hydrophobic drugs are conjugated. In contrast to this approach, the Thiomab technology achieves the desired site-selective and homogeneous modifications on the antibody by exploiting engineered reactive cysteines, not involved in structural disulfide bonding. This approach leads to homogeneous ADCs with defined payload attachment and drug stoichiometry. In general, cysteine mutations are designed to facilitate functionalization with cytotoxic payloads while maintaining the mAb stability, binding affinity and minimizing the ADC aggregation. To identify optimal positions for the mutation, several techniques are used including computational modelling, screening of model system, and high throughput scanning [144,145,146].

Thiomab strategy was described first by Junutula and al. [147]. They reported an anti-MUC16 antibody bearing engineered cysteine residues replacing the heavy chain alanine 114 (HC-A114). The cysteine mutations are formed as mixed disulphides with cysteine or glutathione. To activate them the antibody was treated with TCEP/DTT, and the reduction was found to partially disrupt the interchain disulphides too. After purification the mAb was oxidized using CuSO_4_ or dehydro-ascorbic acid (dhAA) which reformed the interchain bridges. The reactive thiols inside the engineered position were able to react with maleimide bearing linker-payload. The synthesized anti-MUC16 ADC demonstrated in vivo efficacy in a xenograft mouse model and tolerance at higher doses in rats and cynomolgus monkeys. These findings established the Thiomab approach as a general method for bioconjugation.

The cysteine microenvironment can influence the stability of the maleimide attachment, its opening leads to conjugation stability issues and alters the pharmacological properties of the ADC. Shen and coworkers engineered trastuzumab with three different conjugation sites (LC-V205C, HC-A114C, and Fc-S396C) [148]. The introduced cysteine residues had different microenvironments, solvent accessibility and local charge. Fc-396C had the highest solvent exposure compared to the two others mutation sites, which were localized in partially buried regions. The study demonstrated that the high solvent accessibility exposes the linker-payload to reactive thiol groups of plasma albumin, which can lead to maleimide exchange. In contrast, the buried cysteine residues are protected by the steric hindrance thus preventing this phenomenon. Nevertheless, a positive locale charge could accelerate hydrolysis of the succinimide ring in the linker compared to neutral environment. Despite the similar DAR and in vitro activity of the ADCs, the study showed their different stability and therapeutic activity in a mouse tumor model. Hence, the conjugation site affects the stability of the antibody-linker interface, the hydrophobicity of the conjugate, and the ADC performance.

In addition, the succinimide linkage can undergo two parallel reactions in plasma: retro-Michael reaction leading to the loss of the linker-payload, and hydrolysis of the succinimide, both having a significant influence on the ADC activity in vivo. To improve stability, Lyon and collaborators designed linkers that incorporate a basic amino group adjacent to the maleimide [149]. The incorporation of diaminopropionic acid (DPR) inside the linker promoted rapid and quantitative hydrolysis of thiosuccinimides at neutral pH and room temperature. This way the nonspecific deconjugation was prevented leading to increased stability in in vivo studies. Besides the commonly used maleimides, different cysteine-reactive agents were also explored, such as iodoacetamides [150], bromomaleimides [151], carbonylacrylic reagents [152], and *N*-alkyl vinylpyridinium salts [153].

As demonstrated in this section, the Thiomab technology opens access to site-specifically modified, homogeneous ADCs. Though an optimization of the engineered cysteine positions is needed, this approach is up to date one on the most efficient bioconjugation techniques applicable to various linker-payloads that results in stable ADCs with attractive pharmacological properties.

#### 4.2.3. Conjugation to Engineered Unnatural Amino Acids/SelenomAb

In addition to the Thiomab technology, the incorporation on non-canonical amino acids (ncAA) offers another possibility for site-specific conjugation. This technique allows the incorporation of amino acids having a unique chemical structure, which enables the introduction of linker-payload conjugates in a chemoselective manner. The reactive handle is engineered at specific sites in the antibodies allowing the drug conjugation with defined stoichiometry. The technology requires rearrangement of the antibody sequence. To achieve this, a tRNA/aminoacyl-tRNA synthetase (aaRS) pair, that is orthogonal to all the endogenous tRNAs and synthetases in the host cell, is used to incorporate an ncAA into a protein in response to an unassigned codon, typically a stop codon. Normally the ncAA is supplemented to the media during fermentation [154]. The choice of the unnatural amino acid is important because the incorporation could provoke a possible immunogenicity. Commonly used ncAA are analogues of natural amino acids bearing unique functionalities such as ketones, azides, cyclopropenes or diene function.

Schultz and co-workers reported a successful incorporation of p-acetylphenylalanine (pAcF) into an anti-CXCR4 IgG. The payload auristatin was efficiently conjugated through oxime ligation to the antibody, affording a chemically homogeneous ADC. The conjugate displayed excellent in vitro activity and complete eradication of pulmonary tumor lesions in mice [155].

Because of the acidic conditions that are required for oxime ligation, and slow release kinetics from the ADC, an alternative is to incorporate an azide containing ncAA. The widely used para-azidophenylalanine (pAzF) allows to perform rapid CuAAC or SPAAC reactions in physiological conditions. Brandish et al. exploited this strategy on anti-CD74 antibody conjugated to glucocorticoid payload through a diphosphatase-cleavable linker [156]. In addition to the pAcF technique, several azide containing lysine analogues (AzK) have been successfully incorporated in antibodies to generate site-specific ADCs with auristatin, PBD dimer or tubulysin payloads [157,158].

Chin and co-workers demonstrated the successful incorporation of a cyclopropene derivative of lysine (CypK) into antibody [159]. They choose 1,3-disubstituted cyclopropene that provides a minimal bioorthogonal handle for conjugation. CypK undergoes rapid and selective inverse-electron-demand Diels–Alder cycloaddition with tetrazine derivatives. The resulting dihydropyridazines covalently linked the antibody to monomethyl auristatin and the conjugate displayed excellent stability in serum.

In parallel to the above-described strategies, the incorporation of naturally occurring atypical amino acid such as selenocysteine (Sec) was also applied as a method for site-specific conjugation into monoclonal antibodies. These engineered mAbs are known as selenomabs. They have the advantages to undergo rapid, efficient and single step site-selective reactions under near physiological conditions. The conjugation occurs without the need of catalyst or redox treatment of disulphide bridges typically used in the Thiomab strategy. In addition, the selenol group is more nucleophilic than the thiol group and is deprotonated at lower pH which provides specific reactivity for this amino acid. Rader and co-workers exploited the unique reactivity of selenomab by conjugating MMAF to anti-HER2 and anti-CD138 antibody using an iodoacetamide-based linker. They demonstrated that positioning the Sec residue in the CH3 loop of the antibody affords DAR 2 selenomab ADC with excellent stability, selectivity, as well as in vitro and in vivo activity [160].

All the above-mentioned strategies of ncAA incorporation display promising results. The site- specificity of this methodology requires the rearrangement of the antibody sequence but allows to control the exact localization and number of reactive residues. In this way it offers multiple possibilities for selective conjugation in the ADC field joining the fierce competition between site-specific technologies on native and engineered antibodies.

## 5. Overview of the ADCs in Late-Stage Clinical Development

As Section 2, Section 3 and Section 4 have revealed, there is very intense development of all compartments of the ADCs. Chemical modifications enable the expansion of our collection of useful payloads, new linker classes emerge that improve ADC characteristics, and recent developments of the bioconjugation toolbox enable the generation of ADCs having increased homogeneity, stability, and efficiency. It is not surprising that the advances in the three areas fuel the pipeline of ADCs in the clinic. Table 3, Table 4 and Table 5 summarize the ADCs in late clinical development as of February 2021. The ADCs are grouped by the nature of the payload separating “small molecules” (Table 3) from biomacromolecules (Table 4) and radioisotopes (Table 5). Besides their names, the nature of the payload and the linker aare also listed where available, as well as the level of their advancement. The ADCs listed cover the whole range from natural to engineered antibodies, whole antibodies to fragments, cleavable to non-cleavable linkers, and payloads from radionuclei to toxins and enzymes, reflecting the myriad of possibilities in this area, and projecting a bright future of antibody drug conjugates in the treatment of different diseases. The ADCs are listed at the highest phase of development meaning that approved ADCs that have active Phase 3 studies for other indications are listed only in Table 1.

ADCs in late-stage clinical development carrying a bio(macro)molecule as a payload are listed in Table 4. It is important to mention that contrary to the “classical” ADCs listed in Table 3 the majority of the drug candidates in Table 4 don’t contain a full IgG-like antibody but only a single chain peptide fused to the payload.

Table 5 lists such ADCs in late clinical development where the drug part is a radioactive isotope, which exerts its effect through radioactive decay. Besides being used as targeted therapies through radiation induced damage, some of these conjugates also have a diagnostic value. While the radionuclei of metals are connected to the antibody through a chelating group and secondary interactions, the 131-I isotope is usually embedded into the antibodies through the chemical modification (electrophilic iodination) of its Tyr residues.

## 6. Conclusions

Although the first ADC received its first approval from the FDA more than 20 years ago, the pharmaceutical industry had to go through a long and tedious learning process to reach a steady pipeline of ADCs both on the market and in clinical development. In this period chemistry enabled the enrichment of the pool of available payloads by establishing a collection of linking methods to connect drug payloads. This process was also aided by the increasing collection of molecular modelling tools that were adapted to this compound class [161,162]. Medicinal chemists have also studied systematically the relationship between the chemical nature of the link between the antibody and the drug, and the properties of the ADC, which led to an improved design of new ADCs. Finally, the recently developed selective chemical modifications of monoclonal antibodies resulted in an improved control of the ADC composition, which in turn ensures a better control of ADC properties. In spite of this significant progress in the chemistry of antibodies–drugs conjugation modalities there is still a lot to learn and explore regarding the conversion of this knowledge into better antibody–drug conjugates.

## Figures and Tables

**Table 1:** ADCs approved in the USA/EU until 2020.

ADC	Target	Payload	Linker	Indication
gemtuzumab ozogamicin (Mylotarg, **1**)	CD33	Calicheamicin	Cleavable, Hydrazone	Acute myeloid leukemia
brentuximab vedotin (Adcetris, **2**)	CD30	MMAE ^1^	Cleavable, Peptide	Hodgkin leukemia; Systemic anaplastic large-cell lymphoma
trastuzumab emtansine (Kadcyla, **3**)	HER2	DM1	Non-cleavable, Thioether	Breast cancer
inotuzumab ozogamicin (Besponsa, **4**)	CD22	Calicheamicin	Cleavable, Hydrazone	B-cell Acute lymphocytic leukemia
polatuzumab vedotin (Polivy, **5**)	CD79b	MMAE	Cleavable, Peptide	Diffuse large B-cell lymphoma
enfortumab vedotin (Padcev, **6**)	Nectin-4	MMAE	Cleavable, Peptide	Urothelial cancer
trastuzumab deruxtecan (Enhertu, **7**)	HER2	deruxtecan	Cleavable, Peptide	Breast cancer
sacituzumab govitecan (TRODELVY, **8**)	TROP-2	SN-38	Cleavable, Peptide	Breast cancer
belantamab mafodotin (BLENREP, **9**)	BCMA	MMAF	Non-cleavable, Thioether?	Multiple myeloma
moxetumomab pasudotox (Lumoxiti, **10**)	CD22	PE38	Fusion protein	Hairy cell leukemia
tagraxofusp (Elzonris, **11**)	IL-3	Diphteria toxin	Fusion protein	Blastic plasmacytoid dendritic cell neoplasm
ibritumomab tiuxetan (ZEVALIN, **12**)	CD20	90-yttrium	tiuxetan	Multiple hematological disorders

^1^ MMAE: monomethyl auristatin E; DM1: N(2′)-deacetyl-N(2′)-(3-mercapto-1-oxopropyl)- maytansine; HER2: human epidermal growth factor 2; TROP-2: antitrophoblast cell-surface antigen 2; BCMA: B-cell maturation antigen; MMAF: monomethyl auristatin F; PE38: Pseudomonas Aeruginosa exotoxin.

**Table 2:** Examples of Tubulysin-derived payloads linked through a modified phenyl group.

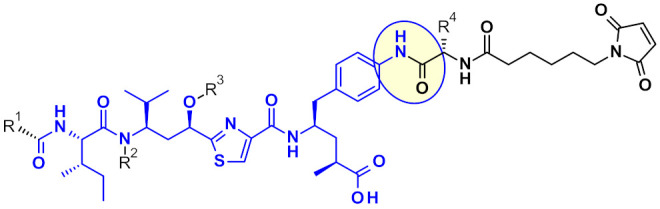				
	R^1^	R^2^	R^3^	R^4^
AstraZeneca	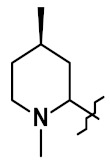	Et	Ac	(CH_2_)_4_NH_2_
Bristol-Myers Squibb	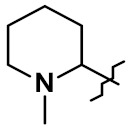	nPr	Me	(CH_2_)_4_NH_2_CONH_2_
Pfizer	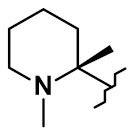	Me	CONHEt	H

**Table 3:** ADCs in late clinical development with a small molecule drug payload and their key features.

ADC Name	Target	Payload	Linker	Phase
BAT8001	HER-2	Maytansinoid	non-cleavable	3
disitamab vedotin	HER-2	MMAE	Val-Cit-PABA	3
DS-1062a	TROP-2	DXd	Gly-Gly-Phe-Gly	3
loncastuximab Tesirine	CD19	SG3199	Val-Ala-PABA	3
mirvetuximab soravtansine	FOLR-α	DM4	sulfo-SPDB ^1^	3
ZRC-3256 ^2^	HER-2	DM1	SMCC ^3^	3
ANG1005 ^4^	LRP1	Paclitaxel	covalent	3
SAR408701	CEACAM5	DM4	SPDB ^5^	3
TAA013	HER-2	DM1	SMCC ^3^	3
trastuzumab duocarmazine	HER-2	seco-DUBA	Val-Cit-PABA	3
tisotumab vedotin	TF	MMAE	Val-Cit-PABA	3
ARX788	HER-2	Amberstatin269	oxime	2/3
ABBV-3373	TNF-α	Steroid	Ala-Ala-PABA	2
anetumab ravtansine	MSLN	DM4	SPDB ^5^	2
BA3011	Axl	MMAE	cleavable	2
camidanlumab Tesirine	CD25	SG3199	Val-Ala-PABA	2
labetuzumab Govitecan	CEACAM5	SN-38	CL2A ^6^	2
ladiratuzumab vedotin	LIV-1	MMAE	Val-Cit	2
MRG003	EGFR	MMAE	Val-Cit-PABA	2
naratuximab emtansine	CD37	DM1	SMCC ^3^	2
patritumab Deruxtecan	HER-3	DXd	Gly-Gly-Phe-Gly	2
praluzatamab ravtansine	CD166	DM4	SPDB ^5^	2
telisotuzumab vedotin	c-MET	MMAE	Val-Cit-PABA	2
VLS-101	ROR1	MMAE	Val-Cit-PABA	2

^1^ 4-thio-2-sulfobutanoyl. ^2^ ZRC-3256 is a generic of **3**. ^3^ 4-(3-thio-*N*-maleimidomethyl)cyclohexane-1-carboxyl. ^4^ the delivery agent is not an antibody but the Angiopep-2 peptide. ^5^ 4-thiobutanoyl. ^6^ SPDB-based PEG containing a cleavable Lys-PABA linker.

**Table 4:** ADCs in late clinical development with a biological macromolecule drug payload and their key features.

ADC Name	Target	Payload	Linker	Phase
cintredekin besudotox ^1^	IL-13R	Pseudomonas exotoxin A	fusion	3
E7777 ^2^	IL-2R	Diptheria Toxin A,B	fusion	3
oportuzumab monatox ^3^	EpCAM	Pseudomonas exotoxin A	fusion	3
T-Guard ^4^	CD7 and CD3	Ricin A	undisclosed	3
naptumomab estafenatox ^5^	5T4	Staphylococcal Enterotoxin E	fusion	2/3
Proxinium ^6^	EpCAM	Pseudomonas exotoxin A	fusion	2/3
EP-100 ^7^	LHRH	CLIP-71	fusion	2
L-DOS47 ^8^	CEACAM6	Urease	SIAB	2
LMB-2 ^9^	CD25	Pseudomonas exotoxin	fusion	2
MDNA55 ^10^	IL-4R	Pseudomonas exotoxin A	fusion	2
MT-3724 ^11^	CD20	Shiga-like toxin A	fusion	2
Resimmune ^12^	CD3	Diphtheria toxin	fusion	2
RO6927005 ^13^	MSLN	Pseudomonas exotoxin A	fusion	2

^1^ fusion protein of IL-13 and a modified toxin. ^2^ fusion protein of IL-2 and modified toxins. ^3^ fusion protein of a single chain fragment of a mAb and a toxin. ^4^ combination of a DD7 targeting ADC and a CD3 targeting ADC. ^5^ fusion protein of the Fab portion of a 5T4 mAb and a toxin. ^6^ fusion protein of a single chain fragment of a mAb and a toxin. ^7^ a LHRH natural ligand (10 amino acids) joined to an 18 amino acid cationic α-helical lytic peptide. ^8^ conjugate of a single domain camelid antibody and Jack bean urease enzyme through a *N*-succinimidyl[4-iodoacetyl] aminobenzoate linker. ^9^ fusion of the variable heavy domain of anti-CD25 and a modified toxin. ^10^ fusion protein of IL-4 and a modified toxin. ^11^ fusion protein of the variable fragment of a mAb and a toxin. ^12^ fusion of a modified toxin with two single chain antibody fragments. ^13^ fusion protein of a Fab mAb fragment and a modified toxin.

**Table 5:** ADCs in late clinical development with a radioisotope as drug payload and their key features.

ADC Name	Target	Payload	Linker	Phase
TLX250-CDx	carbonic anhydrase IX	89-Zr	DOFA ^1^	3
IMMU-107	MUC-1	90-Y	DOTA ^2^	3
Iomab-B	CD45	131-Iodine	direct iodination	3
131I-8H9	B7-H3	131-Iodine	direct iodination	2/3
111In-J591	PSMA	111-In	DOTA ^2^	2
177Lu-DOTA-girentuximab	carbonic anhydrase IX	177-Lu	DOTA ^2^	2
177Lu-DOTA-Rosopatamab	FOLH1	177-Lu	DOTA ^2^	2
Betalutin	CD37	177-Lu	p-SCN-Bn-DOTA	2
CLR 131 ^3^	Lipid raft	131-I	direct iodination	2
64Cu-DOTA-trastuzumab	HER-2	64-Cu	DOTA ^2^	2
111 In-ibritumomab tiuxetan	CD20	111-In	modified-DTPA	2

^1^ deferoxamine. ^2^ tetraxetan. ^3^ phospholipid drug conjugate.

## Data Availability

Not applicable.
